# Utilizing sine trigonometric q-spherical fuzzy rough aggregation operators for group decision-making and their role in digital transformation

**DOI:** 10.1016/j.heliyon.2024.e30758

**Published:** 2024-05-09

**Authors:** Ahmad Bin Azim, Asad Ali, Abdul Samad Khan, Fuad A. Awwad, Emad A.A. Ismail, Sumbal Ali

**Affiliations:** a Department of Mathematics and Statistics, Hazara University Mansehra, 21300, Khyber Pakhtunkhwa, Pakistan; b Research Center for Computational Science, School of Mathematics and Statistics, Northwestern Polytechnical University, Xi'an, 710129, China; c Department of Quantitative Analysis, College of Business Administration, King Saud University, P.O. Box 71115, Riyadh, 11587, Saudi Arabia

**Keywords:** Multiple-criteria decision-making q-spherical fuzzy rough sets, Aggregation operators, Sine trigonometric operations in decision-making

## Abstract

q-spherical fuzzy rough set (q-SFRS) is also one of the fundamental concepts for addressing more uncertainties in decision problems than the existing structures of fuzzy sets, and thus its implementation was more substantial. The well-known sine trigonometric function maintains the periodicity and symmetry of the origin in nature and thus satisfies the expectations of the experts over the multi-parameters. Taking this feature and the significance of the q-SFRSs into consideration, the main objective of the article is to describe some reliable sine trigonometric laws for SFSs. Associated with these laws, we develop new average and geometric aggregation operators to aggregate the q-spherical fuzzy rough numbers. Then, we presented a group decision-making strategy to address the multi-attribute group decision-making problem using the developed aggregation operators. To verify the value of the defined operators, a MAGDM strategy is provided along with applications for selecting a Cloud Service Provider and a Digital Transformation Vendor for digital transformation. Moreover, a comparative study is also performed to present the effectiveness of the developed approach.

## 1 Introduction

Multi-attribute group decision-making (MAGDM) stands as a sophisticated technique designed to navigate decision scenarios involving multiple factors and numerous decision-makers. The inherent complexity of such scenarios necessitates the consideration of various variables, including the preferences and viewpoints of all decision-makers, the significance and applicability of each attribute, and the potential uncertainties that may emerge throughout the decision-making process. Group decision-making (GDM), recognized as a cooperative process involving multiple participants in decision-making, has garnered significant attention across diverse research domains [1,2]. While the adoption of GDM has gained popularity, it introduces challenges, particularly in scenarios where decision-makers grapple with unclear situations and may resort to arbitrary decisions. To address the inherent ambiguities and uncertainties in real-world settings, the concept of fuzzy sets (FS) was introduced [3]. Within this framework, each element is assigned a degree of membership, laying the foundation for the extension known as the intuitionistic fuzzy set (IFS) [4]. IFS introduced degrees of membership and non-membership, governed by the condition ζ + υ ≤ 1 within the range of [0, 1]. Scholars have embraced IFS, contributing algorithms, aggregation operators, and operational laws [5,6]. Further extensions include the Pythagorean fuzzy set (PyFS) [7], characterized by membership and non-membership degree satisfying the condition that the sum of squares of ζ and υ is equal to or less than 1. Additionally, the Fermatean fuzzy set (FFS), proposed by Senapati and Yager [8], and the q-rung orthopair fuzzy set (q-ROFS), introduced by Yager [9], represent generalized formats incorporating IFS, PyFS, and FFS. Despite advancements, existing approaches have limitations, particularly in dealing with various indicators. For instance, when working with a picture fuzzy set, techniques are restricted to the range of μ + υ + ζ ≤ 1. Mehmood et [44]. presented the approach toward decision-making and medical diagnosis problems using the concept of spherical fuzzy sets. Similarly, in the context of a spherical fuzzy set [10], the approach is confined to the range of $\mu^{2}+\upsilon^{2}+\zeta^{2}\leq 1$. In the case of q-SFS [11], the approaches are confined to the domain of $\mu^{q}+\upsilon^{q}+\zeta^{q}\leq 1$. Decision-makers in a q-SFS framework are constrained to assigning equal values of q to the membership, non-membership, and neutral membership degrees during their decision-making processes. Notably, when information is presented in the form of spherical fuzzy rough sets (SFRS), a drawback arises as the total of the lower and upper approximations values exceeds the range [0,1]. Specifically, expressions such as $\left(0⩽̸0.7^{2}+0.8^{2}+0.9^{2}⩽̸1\right)\;\text{and}\;\left(0⩽̸0.9^{2}+0.7^{2}+0.8^{2}⩽̸1\right)$ cannot be effectively handled by SFRS. To address this limitation, Azim et al. [12] proposed the q-spherical fuzzy rough set (q-SFRS), introducing a more comprehensive condition $\left(0\leq 0.7^{q}+0.8^{q}+0.9^{q}\leq 1\right)\;\text{and}\;\left(0\leq 0.9^{q}+0.7^{q}+0.8^{q}\leq 1\right)$ for the q≥1. This increased flexibility empowers decision-makers to adjust their values, enabling more accurate and nuanced conclusions. In essence, q-SFRS provides a more adaptable framework for decision-making in scenarios involving q-spherical fuzzy rough sets. Aggregation operators serve as mathematical tools in multi-criteria decision-making (MCDM), amalgamating several criteria or alternatives for informed decision-making. Prominent aggregation operations include TOPSIS, weighted geometric, and weighted averaging. While these operations play a crucial role in simplifying complex decision-making situations, challenges persist. Azim et al. [13] presented a solution to MCDM difficulties, and subsequent research by Ali et al. [14], Wang et al. [15], Liu and Wang [16], Farid and Fiaz [17], and Garg [18] introduced various aggregation methods and operators within specific contexts. Notably, sine trigonometric aggregation operators (AOs) have been developed by Garg [18] for a range of criteria group decision-making issues under Pythagorean fuzzy sets. Additionally, Riaz et al. [19] described sine trigonometric AOs in a bipolar fuzzy environment. Recognizing the foundational importance of operational laws, the idea of q-spherical fuzzy rough sets and their application in multi-attribute decision-making problems was pioneered by Azim et al. [12]. The ensuing development of the q-SFRS framework, enriched by generalized operational laws, has broadened its applications and enhanced adaptability in various scenarios. These generalized operational laws pave the way for future research and development, enabling q-SFRS to tackle an even greater variety of challenges. Recent research has made significant strides in the field of decision-making methodologies and computational sciences. Noteworthy contributions include studies on the selection of Database Management Systems using a Multi-Attribute Decision-Making Approach based on Probability Complex Fuzzy Aggregation Operators [45], an evaluation of computer networks under the environment of bipolar complex fuzzy partition Heronian mean operators [46], and the development of the MABAC framework for logarithmic bipolar fuzzy multiple attribute group decision-making, particularly applied to supplier selection [47]. Moreover, researchers have proposed advancements in decision-making algorithms, such as an improved interval type-2 fuzzy VIKOR method [48], a T-Spherical Fuzzy Information-based multi-attribute decision-making algorithm utilizing Heronian Mean Operators [49], and a novel approach to multi-attribute group decision-making using Pythagorean Fuzzy Rough Set and the Schweizer-Sklar T-norm and T-conorm [50]. These contributions collectively contribute to the evolving landscape of decision-making theories and computational methodologies, offering valuable insights and tools for addressing complex decision scenarios. The properties of PF t-norm & conorm are examined by Cuong et al. [51]. Ashraf et al. [52,53] using the Dombi method, described some SF aggregation operators and discussed their decision-making application, also studied the presentation of SF t-norm and conorm. Spherical fuzzy sets and their representation of Spherical fuzzy t-norms and t-conorms are discussed by Kutlu Gundogdu and Kahraman [54]. Garg discussed generalized intuitionistic fuzzy interactive geometric interaction operators using Einstein's t-norm and t-conorm and their application to decision-making [55]. Garg also presented Pythagorean fuzzy geometric aggregation operators using Einstein t‐norm and t‐conorm for a multicriteria decision‐making process [56]. Addressing data ambiguity and inherent periodic and symmetrical properties, this study explores the application of trigonometric operations, particularly sine trigonometric (ST) functions, in the context of q-SFRS. The research highlights a significant gap in understanding how ST operations can be effectively applied to q-SFRS, emphasizing the need for tailored operational laws. To bridge this research gap, the study proposes a set of operational principles termed STOL (Sine Trigonometric-based Operational regulations). These STOLs are specifically designed for q-SFRS, aiming to enhance its capabilities in solving complex issues with greater accuracy and dependability. Alongside STOLs, the study introduces aggregation operators (AOs) tailored to specific q-SFRS pairings, systematically utilizing the q-SFRS framework. The primary goals of this work include presenting the STOLs, developing specific AOs for q-SFRS pairings, investigating the connections underlying them, and devising an effective method to address numerous attribute group decision-making (NAGDM) issues. The study concludes with a numerical example demonstrating the efficiency of the proposed method. The application of sine operational laws and their related aggregation operators in multi-criteria decision-making (MCDM) offers a unique way to manage the complexity involved in decision-making processes. These ideas, which are based on the sine function's periodicity and symmetry characteristics, have certain advantages in MCDM scenarios. Aggregation techniques incorporating sine operating principles have various benefits. First, cyclical or repeated patterns may be included while evaluating criteria since the sine function is periodic. This is particularly useful when there are time-dependent criteria and cycles or seasonal oscillations in the decision environment. Furthermore, circumstances where the criteria have equal weight in both positive and negative directions may be handled because of the sine function's symmetrical structure. It is possible to properly evaluate criteria in both favorable and unfavorable results when symmetric aggregating operators are employed. The sine operational laws give rise to aggregation operators, which offer a generic foundation for effectively combining several criteria in MCDM. These operators' ability to identify these fundamental characteristics and the connections between criteria results in decision outputs that are more precise and perceptive.)

The following main goals are the focus of the planned work.

1. Present the STOLs, a unique set of operating laws for the q-spherical fuzzy rough set (q-SFRS).
2. Create aggregation operators (AOs) that are specific to certain q-SFRS pairings and investigate the connections that underlie them.
3. Develop an effective method to handle numerous attribute group decision-making (NAGDM) issues by utilizing the recently introduced STOLs and aggregation operators.
4. Give a numerical example to demonstrate the efficiency of the method.

The arrangement of the study is as follows Section 2 presents a comprehensive summary of q-SFRS, providing an overview of its fundamental concepts and principles. In Section 3, a detailed description of the new STOLs (Sine-Trigonometric Operational Laws) and associated axioms is presented. This section aims to establish a clear understanding of the operational laws that form the basis of the study. Building on the established laws, Section 4 introduces a variety of operators that leverage the new STOLs. This section elaborates on how these operators utilize the defined laws in practical applications. Section 5 delves into the practical application of the developed operators, offering an example of a group decision-making method for problem-solving. The efficacy of this method is demonstrated through a real-world case study, providing insights into its potential and practical implications. In Section 6, the study's results are comprehensively presented. This section discusses the outcomes, findings, and any significant contributions made by the study, summarizing the key takeaways for the reader.

## 2 Basic concepts about q-SFRSs

Definition 1 [12] A q-spherical fuzzy relation R in $U_{1}\times U_{2}$ is a q-spherical fuzzy subset of $U_{1}\times U_{2}$ and is given by

(1) $$R=\left\{\left\langle \left(a,b\right):\mu_{R}\left(a,b\right),\zeta_{R}\left(a,b\right),\upsilon_{\;R}\left(a,b\right)\right\rangle :\left({{\left(\mu_{R}\left(a,b\right)\right)}^{q}+\left(\zeta_{R}\left(a,b\right),\right)}^{q}+{\left(\upsilon_{\;R}\left(a,b\right)\right)}^{q}\right)\leq 1:\forall \;a\in U_{1},b\in U_{2}\right\}$$

where $\mu_{R}:U\to \left[0,1\right]$, $\zeta_{R}:U\to \left[0,1\right]\;\text{and}\;\upsilon_{\;R}:U\to \left[0,1\right]\text{.}$ Equation (1) represents the mathematical structure of a fuzzy set.

The degree of refusal s∈U is defined by $r\left(s\right)=\sqrt{1-{{(\;\mu \left(s\right)}^{q}+\zeta \left(s\right)}^{q}+{\upsilon \left(s\right)}^{q})}$:

Definition 2 [12] Let R be a q-spherical fuzzy relation R on two universes, then we call the triplet $\left(U_{1},U_{2},R\right)$ q-spherical fuzzy rough approximation space.

Definition 3 [12] Let A $\subseteq U_{2}$, then the lower and upper approximation of A with respect to $\left(U_{1},U_{2},R\right)$ is defined by

(2) $$A_{Q}=\left({\underline{A}}_{Q},{\bar{A}}_{Q}\right)=\left\{b,\left\langle \mu_{\;\underline{Q}}\left(b\right),\zeta_{\underline{Q}}\left(b\right),\upsilon_{\;\underline{Q}}\left(b\right),\mu_{\;\bar{Q}}\left(b\right),\zeta_{\;\bar{Q}}\left(b\right),\upsilon_{\;\bar{Q}}\left(b\right)\right\rangle :b\in U_{1}\right\}$$

Where,

$$\mu_{\;\underline{Q}}\left(b\right)={⋀}_{s\in U_{2}}\left\{\mu_{R}\left(a,b\right)⋀\mu_{A}\left(b\right)\right\}$$

$$\zeta_{\underline{Q}}\left(b\right)={⋁}_{s\in U_{2}}\left\{\zeta_{R}\left(a,b\right)⋁\zeta_{A}\left(b\right)\right\}$$

$$\upsilon_{\;\underline{Q}}\left(b\right)={⋁}_{s\in U_{2}}\left\{\upsilon_{\;R}\left(a,b\right)⋁\upsilon_{A}\left(b\right)\right\}$$

$$\mu_{\;\bar{Q}}\left(b\right)={⋁}_{s\in U_{2}}\left\{\mu_{R}^{+}\left(a,b\right)⋁\mu_{A}\left(b\right)\right\}$$

$$\zeta_{\;\bar{Q}}\left(b\right)={⋀}_{s\in U_{2}}\left\{\zeta_{R}\left(a,b\right)⋀\zeta_{A}\left(b\right)\right\}$$

$$\upsilon_{\;\bar{Q}}\left(b\right)={⋀}_{s\in U_{2}}\left\{\upsilon_{\;R}\left(a,b\right)⋀\upsilon_{A}\left(b\right)\right\}\text{,}$$

with the condition that

$$\left\{\begin{array}{c} \left({0\leq \left(\mu_{\;\underline{Q}}\left(b\right)\right)}^{q}+{\left(\zeta_{\underline{Q}}\left(b\right)\right)}^{q}+{\left(\upsilon_{\;\underline{Q}}\left(b\right)\right)}^{q}\leq 1\right)\text{,}\\ \left({0\leq \left(\mu_{\;\bar{Q}}\left(b\right)\right)}^{q}+{\left(\zeta_{\;\bar{Q}}\left(b\right)\right)}^{q}+{\left(\upsilon_{\;\bar{Q}}\left(b\right)\right)}^{q}\leq 1\right) \end{array}\right\}\text{.}$$

Equation (2) represents the mathematical structure of a q-spherical fuzzy rough set. The pair of q-spherical fuzzy sets is then said to represent a q-spherical fuzzy rough set (q-SFRS) if $A_{\underline{Q}}\neq {\;A}_{\;\bar{Q}}$. For simplicity, we write $A_{Q}=\left(A_{\underline{Q}},{\bar{A}}_{Q}\right)$ and the expression $A_{Q}=\left(A_{\underline{Q}},{\;A}_{\;\bar{Q}}\right)$ is called a q-spherical fuzzy rough number. $A_{\text{Qi}\;}$ denotes the collection of all q-SFRNs.

Definition 3 [12] Let ${\;A}_{Q1\;}=\left(\mu_{{\underline{Q}}_{1}},\zeta_{{\underline{Q}}_{1}},\upsilon_{{\underline{Q}}_{1}},\mu_{{\;\bar{Q}}_{1}},\zeta_{{\;\bar{Q}}_{1}},\upsilon_{{\;\bar{Q}}_{1}}\right)$ , $A_{Q2\;}=\left(\mu_{{\underline{Q}}_{2}},\zeta_{{\underline{Q}}_{2}},\upsilon_{{\underline{Q}}_{2}},\mu_{{\;\bar{Q}}_{2}},\zeta_{{\;\bar{Q}}_{2}},\upsilon_{{\;\bar{Q}}_{2}}\right)$ and $A_{Q\;}=\left(\mu_{\underline{Q}},\zeta_{\underline{Q}},\upsilon_{\underline{Q}},\mu_{\;\bar{Q}},\zeta_{\;\bar{Q}},\upsilon_{\;\bar{Q}}\right)$ be any three q-SFRNs, and ω>0, then,

1. $A_{Q1\;}⊕A_{Q2\;}=\left\langle \begin{array}{l} \sqrt[{q}]{\mu_{{\underline{Q}}_{1}}^{q}{+\mu }_{{\underline{Q}}_{2}}^{q}{-\mu }_{{\underline{Q}}_{1}}^{q}\mu_{{\underline{Q}}_{2}}^{q}},{\;\zeta }_{{\underline{Q}}_{1}}^{q}\zeta_{{\underline{Q}}_{2}}^{q},\sqrt[{q}]{\left({1-\mu }_{{\underline{Q}}_{2}}^{q}\upsilon_{{\underline{Q}}_{1}}^{q}+{1-\mu }_{{\underline{Q}}_{1}}^{q}\upsilon_{{\underline{Q}}_{2}}^{q}\right){-\upsilon }_{{\underline{Q}}_{1}}^{q}\upsilon_{{\underline{Q}}_{2}}^{q}},\\ \sqrt[{q}]{\mu_{{\;\bar{Q}}_{1}}^{q}{+\mu }_{{\;\bar{Q}}_{2}}^{q}{-\mu }_{{\;\bar{Q}}_{1}}^{q}\mu_{{\;\bar{Q}}_{2}}^{q}},{\;\zeta }_{{\;\bar{Q}}_{1}}^{q}\zeta_{{\;\bar{Q}}_{2}}^{q},\sqrt[{q}]{\left({1-\mu }_{{\;\bar{Q}}_{2}}^{q}\upsilon_{{\;\bar{Q}}_{1}}^{q}+{1-\mu }_{{\;\bar{Q}}_{1}}^{q}\upsilon_{{\;\bar{Q}}_{1}}^{q}\right){-\upsilon }_{{\;\bar{Q}}_{1}}^{q}\upsilon_{{\;\bar{Q}}_{1}}^{q}} \end{array}\right\rangle$,
2. $A_{Q1\;}⊗A_{Q2\;}=\left\langle \begin{array}{l} \mu_{{\underline{Q}}_{1}}^{q}\mu_{{\underline{Q}}_{2}}^{q}.\sqrt[{q}]{\zeta_{{\underline{Q}}_{1}}^{q}{+\zeta }_{{\underline{Q}}_{2}}^{q}{-\zeta }_{{\underline{Q}}_{1}}^{q}\mu_{{\underline{Q}}_{2}}^{q}},\sqrt[{q}]{\left({1-\zeta }_{{\underline{Q}}_{2}}^{q}\upsilon_{{\underline{Q}}_{1}}^{q}+{1-\zeta }_{{\underline{Q}}_{1}}^{q}\upsilon_{{\underline{Q}}_{2}}^{q}\right){-\upsilon }_{{\underline{Q}}_{1}}^{q}\upsilon_{{\underline{Q}}_{2}}^{q}},\\ \mu_{{\;\bar{Q}}_{1}}^{q}\mu_{{\;\bar{Q}}_{2}}^{q},\sqrt[{q}]{\zeta_{{\;\bar{Q}}_{1}}^{q}{+\zeta }_{{\;\bar{Q}}_{2}}^{q}{-\zeta }_{{\;\bar{Q}}_{1}}^{q}\zeta_{{\;\bar{Q}}_{2}}^{q}},\sqrt[{q}]{\left({1-\zeta }_{{\;\bar{Q}}_{2}}^{q}\upsilon_{{\;\bar{Q}}_{1}}^{q}+{1-\zeta }_{{\;\bar{Q}}_{1}}^{q}\upsilon_{{\;\bar{Q}}_{1}}^{q}\right){-\upsilon }_{{\;\bar{Q}}_{1}}^{q}\upsilon_{{\;\bar{Q}}_{1}}^{q}} \end{array}\right\rangle$,
3. $A_{Q}^{\omega }=\left\langle \begin{array}{l} \mu_{\underline{Q}}^{\omega },\sqrt[{q}]{{1-\left(1-\zeta_{\underline{Q}}^{q}\right)}^{\omega }},\sqrt[{q}]{{\left(1-\zeta_{\underline{Q}}^{q}\right)}^{\omega }-{\left(1-\zeta_{\underline{Q}}^{q}-\upsilon_{\underline{Q}}^{q}\right)}^{\omega }},\\ \mu_{\bar{Q}}^{\omega },\sqrt[{q}]{{1-\left(1-\zeta_{\bar{Q}}^{q}\right)}^{\omega }},\sqrt[{q}]{{\left(1-\zeta_{\bar{Q}}^{q}\right)}^{\omega }-{\left(1-\zeta_{\bar{Q}}^{q}-\upsilon_{\bar{Q}}^{q}\right)}^{\omega }} \end{array}\right\rangle$,
4. ${\omega A}_{Q}=\left\langle \begin{array}{l} \sqrt[{q}]{{1-\left(1-\mu_{\underline{Q}}^{q}\right)}^{\omega }},\zeta_{\underline{Q}}^{\omega },\sqrt[{q}]{{\left(1-\mu_{\underline{Q}}^{q}-\upsilon_{\underline{Q}}^{q}\right)}^{\omega }},\\ \mu_{\bar{Q}}^{\omega },\sqrt[{q}]{{1-\left(1-\zeta_{\bar{Q}}^{q}\right)}^{\omega }},\sqrt[{q}]{{\left(1-\mu_{\bar{Q}}^{q}-\upsilon_{\bar{Q}}^{q}\right)}^{\omega }} \end{array}\right\rangle$,
5. $A_{Q1\;}=A_{Q2\;}$ if and only if $\mu_{{\underline{Q}}_{1}}=\mu_{{\underline{Q}}_{2}},\mu_{{\underline{Q}}_{1}}=\mu_{{\underline{Q}}_{2}}$ $\zeta_{{\underline{Q}}_{1}}=\zeta_{{\underline{Q}}_{2}}$ and $\upsilon_{{\underline{Q}}_{1}}=\upsilon_{{\underline{Q}}_{2}}$.

Definition 4 [12] Let $A_{Q\;}=\left(\mu_{\underline{Q}},\zeta_{\underline{Q}},\upsilon_{\underline{Q}},\mu_{\;\bar{Q}},\zeta_{\;\bar{Q}},\upsilon_{\;\bar{Q}}\right)$ be a q-SFRN. Then the score value which is denoted as $A_{Q\;}$ can be determined by the following function.

(3) $$Sco\left(A_{Q\;}\right)=\frac{{2+\left(\mu_{\;\underline{Q}}\right)}^{q}+{\left(\mu_{\;\bar{Q}}\right)}^{q}-{\left(\zeta_{\underline{Q}}\right)}^{q}-{\left(\zeta_{\;\bar{Q}}\right)}^{q}-{\left(\upsilon_{\;\underline{Q}}\right)}^{q}-{\left(\upsilon_{\;\bar{Q}}\right)}^{q}}{3}$$

Where.

0≼Sco(A)≼1. Equation (3) represents the score function od a q-spherical fuzzy rough set.

Definition 5 [12] Let $A_{Q\;}=\left(\mu_{\underline{Q}},\zeta_{\underline{Q}},\upsilon_{\underline{Q}},\mu_{\;\bar{Q}},\zeta_{\;\bar{Q}},\upsilon_{\;\bar{Q}}\right)$ be a q-SFRN. The accuracy of $A_{Q\;}$ is calculated by using the formula mentioned in Equation No. 4.

(4) $$Acc\left(A_{Q\;}\right)=\frac{{\left(\mu_{\;\underline{Q}}\right)}^{q}+{\left(\mu_{\;\bar{Q}}\right)}^{q}-{\left(\upsilon_{\;\underline{Q}}\right)}^{q}-{\left(\upsilon_{\;\bar{Q}}\right)}^{q}}{2}$$

where $-1≼Acc\left(A_{Q\;}\right)≼1$. Equation (4) represents the accuracy function od a q-spherical fuzzy rough set.

Definition 6 [12] Let ${\;A}_{Q1\;}=\left(\mu_{{\underline{Q}}_{1}},\zeta_{{\underline{Q}}_{1}},\upsilon_{{\underline{Q}}_{1}},\mu_{{\;\bar{Q}}_{1}},\zeta_{{\;\bar{Q}}_{1}},\upsilon_{{\;\bar{Q}}_{1}}\right)$ and $A_{Q2\;}=\left(\mu_{{\underline{Q}}_{2}},\zeta_{{\underline{Q}}_{2}},\upsilon_{{\underline{Q}}_{2}},\mu_{{\;\bar{Q}}_{2}},\zeta_{{\;\bar{Q}}_{2}},\upsilon_{{\;\bar{Q}}_{2}}\right)$ are two q-SFRNs, then

1. If $Sco\left(A_{Q1\;}\right)≺Sco\left(A_{Q2\;}\right)$ then $A_{Q1\;}≺A_{Q2\;}$,
2. If $Sco\left(A_{Q1\;}\right)≻Sco\left(A_{Q2\;}\right)$ then $A_{Q1\;}≻A_{Q2\;}$,
3. If $Sco\left(A_{Q1\;}\right)=Sco\left(A_{Q2\;}\right)$ then
  • If $Acc\left(A_{Q1\;}\right)≺Acc\left(A_{Q2\;}\right)$ then $A_{Q1\;}≺A_{Q2\;}$,
  • If $Acc\left(A_{Q1\;}\right)≻Acc\left(A_{Q2\;}\right)$ then $A_{Q1\;}≻A_{Q2\;}$,
  • If $Acc\left(A_{Q1\;}\right)=Acc\left(A_{Q2\;}\right)$ then $A_{Q1\;}=A_{Q2.\;}$

Definition 7 [12] Let ${\;A}_{Q1\;}=\left(\mu_{{\underline{Q}}_{1}},\zeta_{{\underline{Q}}_{1}},\upsilon_{{\underline{Q}}_{1}},\mu_{{\;\bar{Q}}_{1}},\zeta_{{\;\bar{Q}}_{1}},\upsilon_{{\;\bar{Q}}_{1}}\right)$ , $=\left(\mu_{{\underline{Q}}_{2}},\zeta_{{\underline{Q}}_{2}},\upsilon_{{\underline{Q}}_{2}},\mu_{{\;\bar{Q}}_{2}},\zeta_{{\;\bar{Q}}_{2}},\upsilon_{{\;\bar{Q}}_{2}}\right)$ and $A_{Q\;}=\left(\mu_{\underline{Q}},\zeta_{\underline{Q}},\upsilon_{\underline{Q}},\mu_{\;\bar{Q}},\zeta_{\;\bar{Q}},\upsilon_{\;\bar{Q}}\right)$ be any three q-SFRNs, and ω, $\omega_{1}$ and $\omega_{2}$ are any positive integers then the following properties are held.

1. $A_{Q1\;}⊕A_{Q2\;}=A_{Q2\;}⊕A_{Q1\;}$,
2.$A_{Q1\;}⊗A_{Q2\;}=A_{Q2\;}⊗A_{Q1\;}$,
3. $\omega \left(A_{Q1\;}⊕A_{Q2\;}\right)=\omega A_{Q1\;}⊕\omega A_{Q2\;}$,
4. $\omega_{1}A_{Q\;}⊕\omega_{2}A_{Q\;}=\left(\omega_{1}+\omega_{2}\right)A_{Q\;}$,
5. ${\left(A_{Q1\;}⊗A_{Q2}\right)}^{\omega }=A_{Q1}^{\omega }⊗A_{Q2}^{\omega }$,
6. ${A_{Q\;}}^{\omega_{1}}⊗{A_{Q\;}}^{\omega_{2}}={A_{Q\;}}^{\omega_{1}+\omega_{2}}$.

## 3 New operational laws for q-SFRNs

Because it is symmetrical and periodic, the sine trigonometric function may be used in a variety of ways to support decision experts in their assessment of items. Consequently, we have created operating laws for q-SFRNs, or STOLs. Additionally, we have carefully investigated and evaluated several characteristics related to these STOLs. The sine function was selected because of its unique properties, which successfully convey the ambiguity and intrinsic uncertainty in the q-spherical fuzzy rough framework decision-making process. We provide STOLs in this section, which may be used to merge q-SFRNs over X.

For $q-\text{SFRNs},Q=\left\{x,\left\langle \mu_{\;\underline{Q}}\left(x\right),\zeta_{\underline{Q}}\left(x\right),\upsilon_{\;\underline{Q}}\left(x\right),\mu_{\;\bar{Q}}\left(x\right),\zeta_{\;\bar{Q}}\left(x\right),\upsilon_{\;\bar{Q}}\left(x\right)\right\rangle |x\in X\right\}$ the terms $\mu_{\;\underline{Q}}\left(x\right),\zeta_{\underline{Q}}\left(x\right),{\;\upsilon }_{\;\underline{Q}}\left(x\right),\mu_{\;\bar{Q}}\left(x\right),{\;\zeta }_{\;\bar{Q}}\left(x\right)\;\text{and}\;\upsilon_{\;\bar{Q}}\left(x\right)$ satisfy:

1.$\mu_{\;\underline{Q}}\left(x\right):X⟶\left[0,1\right],\zeta_{\underline{Q}}\left(x\right):X⟶\left[0,1\right],\upsilon_{\;\underline{Q}}\left(x\right):X⟶\left[0,1\right],\mu_{\;\bar{Q}}\left(x\right):X⟶\left[0,1\right],\zeta_{\;\bar{Q}}\left(x\right):X⟶\left[0,1\right]and\;\upsilon_{\;\bar{Q}}\left(x\right):X⟶\left[0,1\right]$
2. $\left\{\begin{array}{c} \left({0\leq \left(\mu_{\;\underline{Q}}\left(x\right)\right)}^{q}+{\left(\zeta_{\underline{Q}}\left(x\right)\right)}^{q}+{\left(\upsilon_{\;\underline{Q}}\left(x\right)\right)}^{q}\leq 1\right)\text{,}\\ \left({0\leq \left(\mu_{\;\bar{Q}}\left(x\right)\right)}^{q}+{\left(\zeta_{\;\bar{Q}}\left(x\right)\right)}^{q}+{\left(\upsilon_{\;\bar{Q}}\left(x\right)\right)}^{q}\leq 1\right) \end{array}\right\}\text{,}$ for all x∈X.

The following functions because sine is positive in the first quadrant are:

$$\sin\left(\frac{\pi }{2}\mu_{\;\underline{Q}}\right):X\to \left[0,1\right],\text{for}\;\text{all}\;x\in X\to \sin\left(\frac{\pi }{2}\mu_{\;\underline{Q}}\left(x\right)\right)\in \left[0,1\right]\text{,}$$

$$\sin\left(\frac{\pi }{2}\mu_{\;\bar{Q}}\right):X\to \left[0,1\right],\text{for}\;\text{all}\;x\in X\to \sin\left(\frac{\pi }{2}\mu_{\;\bar{Q}}\left(x\right)\right)\in \left[0,1\right]\text{,}$$

$$\sqrt[{q}]{1-\sin^{q}\left(\frac{\pi }{2}\sqrt[{q}]{1-\zeta_{\;\underline{Q}}^{q}}\right)}:X\to \left[0,1\right]\;\text{for}\;\text{all}\;x\in X\to \sqrt[{q}]{1-\sin^{q}\left(\frac{\pi }{2}\sqrt[{q}]{1-\zeta_{\underline{Q}}^{q}\left(x\right)}\right)}\in \left[0,1\right]\text{,}$$

$$\sqrt[{q}]{1-\sin^{q}\left(\frac{\pi }{2}\sqrt[{q}]{1-\zeta_{\;\bar{Q}}^{q}}\right)}:X\to \left[0,1\right]\;\text{for}\;\text{all}\;x\in X\to \sqrt[{q}]{1-\sin^{q}\left(\frac{\pi }{2}\sqrt[{q}]{1-\zeta_{\;\bar{Q}}^{q}\left(x\right)}\right)}\in \left[0,1\right]\text{,}$$

$$\sqrt[{q}]{1-\sin^{q}\left(\frac{\pi }{2}\sqrt[{q}]{1-\upsilon_{\;\underline{Q}}^{q}}\right)}:X\to \left[0,1\right]\;\text{for}\;\text{all}\;x\in X\to \sqrt[{q}]{1-\sin^{q}\left(\frac{\pi }{2}\sqrt[{q}]{1-\upsilon_{\underline{Q}}^{q}\left(x\right)}\right)}\in \left[0,1\right]$$

and $\sqrt[{q}]{1-\sin^{q}\left(\frac{\pi }{2}\sqrt[{q}]{1-\upsilon_{\;\bar{Q}}^{q}}\right)}:X\to \left[0,1\right]$ for all $x\in X\to \sqrt[{q}]{1-\sin^{q}\left(\frac{\pi }{2}\sqrt[{q}]{1-\upsilon_{\bar{Q}}^{q}\left(x\right)}\right)}\in \left[0,1\right]$ holds. Thus, using this data, we can create a new operator named ST-q-SFRS in the way as follows.

Definition 7 For q-SFRS, $Q=\left\{x,\left\langle \mu_{\;\underline{Q}}\left(x\right),\zeta_{\underline{Q}}\left(x\right),\upsilon_{\;\underline{Q}}\left(x\right),\mu_{\;\bar{Q}}\left(x\right),\zeta_{\;\bar{Q}}\left(x\right),\upsilon_{\;\bar{Q}}\left(x\right)\right\rangle |x\in X\right\}$, ST −q-SFRS of Q is defined as

(5) $$\sin\;Q=\left\{\left(\begin{array}{c} x,\sin\left(\frac{\pi }{2}\mu_{\;\underline{Q}}\right),\sqrt[{q}]{1-\sin^{q}\left(\frac{\pi }{2}\sqrt[{q}]{1-\zeta_{\;\underline{Q}}^{q}}\;\right)},\sqrt[{q}]{1-\sin^{q}\left(\frac{\pi }{2}\sqrt[{q}]{1-\upsilon_{\underline{Q}}^{q}\left(k\right)}\right)}\\ \sin\left(\frac{\pi }{2}\mu_{\;\bar{Q}}\right),\sqrt[{q}]{1-\sin^{q}\left(\frac{\pi }{2}\sqrt[{q}]{1-\zeta_{\;\bar{Q}}^{q}}\;\right)},\sqrt[{q}]{1-\sin^{q}\left(\frac{\pi }{2}\sqrt[{q}]{1-\upsilon_{\bar{Q}}^{q}\left(k\right)}\right)} \end{array}\right):x\in X\right\}$$

which is also q-SFRS. Equation (5) represents the mathematical structure of a sine trigonometric q-spherical fuzzy rough set.

Definition 8 For $A_{Q\;}=\left(\mu_{\underline{Q}},\zeta_{\underline{Q}},\upsilon_{\underline{Q}},\mu_{\;\bar{Q}},\zeta_{\;\bar{Q}},\upsilon_{\;\bar{Q}}\right)$, the number

(6) $$\sin\;Q=\left(\begin{array}{c} \sin\left(\frac{\pi }{2}\mu_{\;\underline{Q}}\right),\sqrt[{q}]{1-{sin}^{q}\left(\frac{\pi }{2}\sqrt[{q}]{1-\zeta_{\;\underline{Q}}^{q}}\;\right)},\sqrt[{q}]{1-\sin^{q}\left(\frac{\pi }{2}\sqrt[{q}]{1-\upsilon_{\underline{Q}}^{q}\left(k\right)}\right),}\\ \sin\left(\frac{\pi }{2}\mu_{\;\bar{Q}}\right),\sqrt[{q}]{1-\sin^{q}\left(\frac{\pi }{2}\sqrt[{q}]{1-\zeta_{\;\bar{Q}}^{q}}\;\right)},\sqrt[{q}]{1-\sin^{q}\left(\frac{\pi }{2}\sqrt[{q}]{1-\upsilon_{\bar{Q}}^{q}\left(k\right)}\right)} \end{array}\right)$$

is also sine trigonometric q-SFRN (ST-q-SFRN). Equation (5) represents the mathematical structure of a sine trigonometric q-spherical fuzzy rough number.

Theorem 1 *Let*$A_{Q\;}=\left(\mu_{\underline{Q}},\zeta_{\underline{Q}},\upsilon_{\underline{Q}},\mu_{\;\bar{Q}},\zeta_{\;\bar{Q}},\upsilon_{\;\bar{Q}}\right)$, *then the function which is defined in Definition* 8 *is also a* q*-SFRN*.

Proof *Straight forward*.

Theorem 2 *Let*${\;A}_{Q1\;}=\left(\mu_{{\underline{Q}}_{1}},\zeta_{{\underline{Q}}_{1}},\upsilon_{{\underline{Q}}_{1}},\mu_{{\;\bar{Q}}_{1}},\zeta_{{\;\bar{Q}}_{1}},\upsilon_{{\;\bar{Q}}_{1}}\right)$*and*$A_{Q2\;}=\left(\mu_{{\underline{Q}}_{2}},\zeta_{{\underline{Q}}_{2}},\upsilon_{{\underline{Q}}_{2}},\mu_{{\;\bar{Q}}_{2}},\zeta_{{\;\bar{Q}}_{2}},\upsilon_{{\;\bar{Q}}_{2}}\right)$*be any two q-SFRNS then we have*

1. $\sin\;{\;A}_{Q1\;}⊕\sin\;{\;A}_{Q2\;}=\sin\;{\;A}_{Q2\;}⊕\sin\;{\;A}_{Q1\;}$.
2. $\sin\;{\;A}_{Q1\;}⊗\sin\;{\;A}_{Q2\;}=\sin\;{\;A}_{Q2\;}⊗\sin\;{\;A}_{Q1\;}$.

Proof Deducible from Definition 8.

Theorem 3 For any three q-SFRNs ${\;A}_{Q1\;}=\left(\mu_{{\underline{Q}}_{1}},\zeta_{{\underline{Q}}_{1}},\upsilon_{{\underline{Q}}_{1}},\mu_{{\;\bar{Q}}_{1}},\zeta_{{\;\bar{Q}}_{1}},\upsilon_{{\;\bar{Q}}_{1}}\right)$, $A_{Q2\;}=\left(\mu_{{\underline{Q}}_{2}},\zeta_{{\underline{Q}}_{2}},\upsilon_{{\underline{Q}}_{2}},\mu_{{\;\bar{Q}}_{2}},\zeta_{{\;\bar{Q}}_{2}},\upsilon_{{\;\bar{Q}}_{2}}\right)$ and

$A_{Q3\;}=\left(\mu_{{\underline{Q}}_{3}},\zeta_{{\underline{Q}}_{3}},\upsilon_{{\underline{Q}}_{3}},\mu_{{\;\bar{Q}}_{3}},\zeta_{{\;\bar{Q}}_{3}},\upsilon_{{\;\bar{Q}}_{3}}\right)$ we have

1. $\left(\sin\;{\;A}_{Q1\;}⊕\sin\;{\;A}_{Q2\;}\right)⊕\sin\;{\;A}_{Q3\;}=\sin\;{\;A}_{Q1\;}⊕\left(\sin\;{\;A}_{Q2\;}⊕\sin\;{\;A}_{Q3\;}\right)$.
2. $\left(\sin\;{\;A}_{Q1\;}⊗\sin\;{\;A}_{Q2\;}\right)⊗\sin\;{\;A}_{Q3\;}=\sin\;{\;A}_{Q1\;}⊗\left(\sin\;{\;A}_{Q2\;}⊗\sin\;{\;A}_{Q3\;}\right)$.

Proof Easily deducible from Definition 8.

Theorem 4 Let ${\;A}_{Q1\;}=\left(\mu_{{\underline{Q}}_{1}},\zeta_{{\underline{Q}}_{1}},\upsilon_{{\underline{Q}}_{1}},\mu_{{\;\bar{Q}}_{1}},\zeta_{{\;\bar{Q}}_{1}},\upsilon_{{\;\bar{Q}}_{1}}\right)$, $A_{Q2\;}=\left(\mu_{{\underline{Q}}_{2}},\zeta_{{\underline{Q}}_{2}},\upsilon_{{\underline{Q}}_{2}},\mu_{{\;\bar{Q}}_{2}},\zeta_{{\;\bar{Q}}_{2}},\upsilon_{{\;\bar{Q}}_{2}}\right)$ and $A_{Q\;}=\left(\mu_{\underline{Q}},\zeta_{\underline{Q}},\upsilon_{\underline{Q}},\mu_{\;\bar{Q}},\zeta_{\;\bar{Q}},\upsilon_{\;\bar{Q}}\right)$ be any three q-SFRNs, and given three real numbers, ω, $\omega_{1}$ and $\omega_{2}$, then the following operational laws are true.

$$\omega \left(\sin\;{\;A}_{Q1\;}⊕\sin\;{\;A}_{Q2\;}\right)=\omega \;\sin\;{\;A}_{Q1\;}⊕\omega \;\sin\;{\;A}_{Q2}\text{.}$$

1. ${\left(\sin\;{\;A}_{Q1\;}⊗\sin\;{\;A}_{Q2\;}\right)}^{\omega }={\left(\sin\;{\;A}_{Q1\;}\right)}^{\omega }⊗{\left(\sin\;{\;A}_{Q2\;}\right)}^{\omega }$.
2. $\omega_{1}\;\sin\;A_{Q\;}⊕\omega_{2}\;\sin\;A_{Q\;}=\left(\omega_{1}+\omega_{2}\right)\sin\;A_{Q\;}$.
3. ${\left(\sin\;A_{Q\;}\right)}^{\omega_{1}}⊗{\left(\sin\;A_{Q\;}\right)}^{\omega_{2}}={\left(\sin\;A_{Q\;}\right)}^{\omega_{2}+\omega_{2}}$.
4. ${\left({\left(\sin\;A_{Q\;}\right)}^{\omega_{1}}\right)}^{\omega_{2}}={\left(\sin\;A_{Q\;}\right)}^{\omega_{1}\omega_{2}}$.

Proof In this demonstration, we will establish the proof for 1 and 3, noting that the other proofs follow a similar approach.

Let ${\;A}_{Q1\;}=\left(\mu_{{\underline{Q}}_{1}},\zeta_{{\underline{Q}}_{1}},\upsilon_{{\underline{Q}}_{1}},\mu_{{\;\bar{Q}}_{1}},\zeta_{{\;\bar{Q}}_{1}},\upsilon_{{\;\bar{Q}}_{1}}\right)$, $A_{Q2\;}=\left(\mu_{{\underline{Q}}_{2}},\zeta_{{\underline{Q}}_{2}},\upsilon_{{\underline{Q}}_{2}},\mu_{{\;\bar{Q}}_{2}},\zeta_{{\;\bar{Q}}_{2}},\upsilon_{{\;\bar{Q}}_{2}}\right)$, then by Definition 8, we have

$$\sin\;{\;A}_{Q1\;}=\left(\begin{array}{c} \sin\left(\frac{\pi }{2}\mu_{\;\underline{Q1}}\right),\sqrt[{q}]{1-\sin^{q}\left(\frac{\pi }{2}\sqrt[{q}]{1-\zeta_{\;\underline{Q1}}^{q}}\;\right)},\sqrt[{q}]{1-\sin^{q}\left(\frac{\pi }{2}\sqrt[{q}]{1-\upsilon_{\underline{Q1}}^{q}\left(k\right)}\right),}\\ \sin\left(\frac{\pi }{2}\mu_{\;\bar{Q1}}\right),\sqrt[{q}]{1-\sin^{q}\left(\frac{\pi }{2}\sqrt[{q}]{1-\zeta_{\;\bar{Q1}}^{q}}\;\right)},\sqrt[{q}]{1-\sin^{q}\left(\frac{\pi }{2}\sqrt[{q}]{1-\upsilon_{\bar{Q1}}^{q}\left(k\right)}\right)} \end{array}\right)\text{and}$$

$$\mathrm{Sin}\;{\;A}_{Q2\;}=\left(\begin{array}{c} \sin\left(\frac{\pi }{2}\mu_{\;\underline{Q2}}\right),\sqrt[{q}]{1-\sin^{q}\left(\frac{\pi }{2}\sqrt[{q}]{1-\zeta_{\;\underline{Q2}}^{q}}\;\right)},\sqrt[{q}]{1-\sin^{q}\left(\frac{\pi }{2}\sqrt[{q}]{1-\upsilon_{\underline{Q2}}^{q}\left(k\right)}\right),}\\ \sin\left(\frac{\pi }{2}\mu_{\;\bar{Q2}}\right),\sqrt[{q}]{1-\sin^{q}\left(\frac{\pi }{2}\sqrt[{q}]{1-\zeta_{\;\bar{Q2}}^{q}}\;\right)},\sqrt[{q}]{1-\sin^{q}\left(\frac{\pi }{2}\sqrt[{q}]{1-\upsilon_{\bar{Q2}}^{q}\left(k\right)}\right)} \end{array}\right)$$

By utilizing the addition laws of q-SFRNs, we obtain

$$\sin\;{\;A}_{Q1\;}⊕\sin\;{\;A}_{Q2\;}=\left(\begin{array}{l} \begin{array}{c} \sqrt[{q}]{1-\left(1-\sin^{p}\left(\frac{\pi }{2}\mu_{\;\underline{Q1}}\right)\right)\left(1-\sin^{p}\left(\mu_{\;\underline{Q2}}\right)\right)},\\ \sqrt[{q}]{1-\sin^{q}\left(\frac{\pi }{2}\sqrt[{q}]{1-\zeta_{{\underline{Q}}_{1}}^{q}}\right)}\sqrt[{q}]{1-\sin^{q}\left(\frac{\pi }{2}\sqrt[{q}]{1-\zeta_{{\underline{Q}}_{2}}^{q}}\right)},\sqrt[{q}]{1-\sin^{q}\left(\frac{\pi }{2}\sqrt[{q}]{1-\upsilon_{{\underline{Q}}_{1}}^{q}}\right)}\sqrt[{q}]{1-\sin^{q}\left(\frac{\pi }{2}\sqrt[{q}]{1-\upsilon_{{\underline{Q}}_{2}}^{q}}\right)}, \end{array}\\ \begin{array}{c} \sqrt[{q}]{1-\left(1-\sin^{p}\left(\frac{\pi }{2}\mu_{\;{\;\bar{Q}}_{1}}\right)\right)\left(1-\sin^{p}\left(\mu_{\;{\;\bar{Q}}_{2}}\right)\right)},\\ \sqrt[{q}]{1-\sin^{q}\left(\frac{\pi }{2}\sqrt[{q}]{1-\zeta_{{\;\bar{Q}}_{1}}^{q}}\right)}\sqrt[{q}]{1-\sin^{q}\left(\frac{\pi }{2}\sqrt[{q}]{1-\zeta_{{\;\bar{Q}}_{2}}^{q}}\right)},\sqrt[{q}]{1-\sin^{q}\left(\frac{\pi }{2}\sqrt[{q}]{1-\upsilon_{{\;\bar{Q}}_{1}}^{q}}\right)}\sqrt[{q}]{1-\sin^{q}\left(\frac{\pi }{2}\sqrt[{q}]{1-\upsilon_{{\;\bar{Q}}_{2}}^{q}}\right)} \end{array} \end{array}\right)$$

$$=\left(\begin{array}{l} \begin{array}{c} \sqrt[{q}]{1-\left(1-\sin^{p}\left(\frac{\pi }{2}\mu_{\;\underline{Q2}}\right)\right)\left(1-\sin^{p}\left(\mu_{\;\underline{Q1}}\right)\right)},\\ \sqrt[{q}]{1-\sin^{q}\left(\frac{\pi }{2}\sqrt[{q}]{1-\zeta_{{\underline{Q}}_{2}}^{q}}\right)}\sqrt[{q}]{1-\sin^{q}\left(\frac{\pi }{2}\sqrt[{q}]{1-\zeta_{{\underline{Q}}_{1}}^{q}}\right)},\sqrt[{q}]{1-\sin^{q}\left(\frac{\pi }{2}\sqrt[{q}]{1-\upsilon_{{\underline{Q}}_{2}}^{q}}\right)}\sqrt[{q}]{1-\sin^{q}\left(\frac{\pi }{2}\sqrt[{q}]{1-\upsilon_{{\underline{Q}}_{1}}^{q}}\right)}, \end{array}\\ \begin{array}{c} \sqrt[{q}]{1-\left(1-\sin^{p}\left(\frac{\pi }{2}\mu_{\;{\;\bar{Q}}_{2}}\right)\right)\left(1-\sin^{p}\left(\mu_{\;{\;\bar{Q}}_{1}}\right)\right)},\\ \sqrt[{q}]{1-\sin^{q}\left(\frac{\pi }{2}\sqrt[{q}]{1-\zeta_{{\;\bar{Q}}_{2}}^{q}}\right)}\sqrt[{q}]{1-\sin^{q}\left(\frac{\pi }{2}\sqrt[{q}]{1-\zeta_{{\;\bar{Q}}_{1}}^{q}}\right)},\sqrt[{q}]{1-\sin^{q}\left(\frac{\pi }{2}\sqrt[{q}]{1-\upsilon_{{\;\bar{Q}}_{2}}^{q}}\right)}\sqrt[{q}]{1-\sin^{q}\left(\frac{\pi }{2}\sqrt[{q}]{1-\upsilon_{{\;\bar{Q}}_{1}}^{q}}\right)} \end{array} \end{array}\right)=\sin\;{\;A}_{Q2\;}⊕\sin\;{\;A}_{Q1}\text{.}$$

*For*ω≻0, *we have*

$$\omega \left(\sin\;{\;A}_{Q1\;}⊕\sin\;{\;A}_{Q2\;}\right)=\left(\begin{array}{c} \begin{array}{c} \sqrt[{q}]{1-{\left(1-\sin^{p}\left(\frac{\pi }{2}\mu_{\;\underline{Q1}}\right)\right)}^{\omega }{\left(1-\sin^{p}\left(\mu_{\;\underline{Q2}}\right)\right)}^{\omega }},\\ {\left(\sqrt[{q}]{1-\sin^{q}\left(\frac{\pi }{2}\sqrt[{q}]{1-\zeta_{{\underline{Q}}_{1}}^{q}}\right)}\sqrt[{q}]{1-\sin^{q}\left(\frac{\pi }{2}\sqrt[{q}]{1-\zeta_{{\underline{Q}}_{2}}^{q}}\right)}\right)}^{\omega },{\left(\sqrt[{q}]{1-\sin^{q}\left(\frac{\pi }{2}\sqrt[{q}]{1-\upsilon_{{\underline{Q}}_{1}}^{q}}\right)}\sqrt[{q}]{1-\sin^{q}\left(\frac{\pi }{2}\sqrt[{q}]{1-\upsilon_{{\underline{Q}}_{2}}^{q}}\right)}\right)}^{\omega }, \end{array}\\ \begin{array}{c} \sqrt[{q}]{1-{\left(1-\sin^{p}\left(\frac{\pi }{2}\mu_{\;{\;\bar{Q}}_{1}}\right)\right)}^{\omega }{\left(1-\sin^{p}\left(\mu_{\;{\;\bar{Q}}_{2}}\right)\right)}^{\omega }},\\ {\left(\sqrt[{q}]{1-\sin^{q}\left(\frac{\pi }{2}\sqrt[{q}]{1-\zeta_{{\;\bar{Q}}_{1}}^{q}}\right)}\sqrt[{q}]{1-\sin^{q}\left(\frac{\pi }{2}\sqrt[{q}]{1-\zeta_{{\;\bar{Q}}_{2}}^{q}}\right)}\right)}^{\omega },{\left(\sqrt[{q}]{1-\sin^{q}\left(\frac{\pi }{2}\sqrt[{q}]{1-\upsilon_{{\;\bar{Q}}_{1}}^{q}}\right)}\sqrt[{q}]{1-\sin^{q}\left(\frac{\pi }{2}\sqrt[{q}]{1-\upsilon_{{\;\bar{Q}}_{2}}^{q}}\right)}\right)}^{\omega } \end{array} \end{array}\right)=\left(\begin{array}{c} \begin{array}{c} \sqrt[{q}]{1-{\left(1-\sin^{p}\left(\frac{\pi }{2}\mu_{\;\underline{Q1}}\right)\right)}^{\omega }{\left(1-\sin^{p}\left(\mu_{\;\underline{Q2}}\right)\right)}^{\omega }},\\ {\left(\sqrt[{q}]{1-\sin^{q}\left(\frac{\pi }{2}\sqrt[{q}]{1-\zeta_{{\underline{Q}}_{1}}^{q}}\right)}\sqrt[{q}]{1-\sin^{q}\left(\frac{\pi }{2}\sqrt[{q}]{1-\zeta_{{\underline{Q}}_{2}}^{q}}\right)}\right)}^{\omega },{\left(\sqrt[{q}]{1-\sin^{q}\left(\frac{\pi }{2}\sqrt[{q}]{1-\upsilon_{{\underline{Q}}_{1}}^{q}}\right)}\sqrt[{q}]{1-\sin^{q}\left(\frac{\pi }{2}\sqrt[{q}]{1-\upsilon_{{\underline{Q}}_{2}}^{q}}\right)}\right)}^{\omega }, \end{array}\\ \begin{array}{c} \sqrt[{q}]{1-{\left(1-\sin^{p}\left(\frac{\pi }{2}\mu_{\;{\;\bar{Q}}_{1}}\right)\right)}^{\omega }{\left(1-\sin^{p}\left(\mu_{\;{\;\bar{Q}}_{2}}\right)\right)}^{\omega }},\\ {\left(\sqrt[{q}]{1-\sin^{q}\left(\frac{\pi }{2}\sqrt[{q}]{1-\zeta_{{\;\bar{Q}}_{1}}^{q}}\right)}\sqrt[{q}]{1-\sin^{q}\left(\frac{\pi }{2}\sqrt[{q}]{1-\zeta_{{\;\bar{Q}}_{2}}^{q}}\right)}\right)}^{\omega },{\left(\sqrt[{q}]{1-\sin^{q}\left(\frac{\pi }{2}\sqrt[{q}]{1-\upsilon_{{\;\bar{Q}}_{1}}^{q}}\right)}\sqrt[{q}]{1-\sin^{q}\left(\frac{\pi }{2}\sqrt[{q}]{1-\upsilon_{{\;\bar{Q}}_{2}}^{q}}\right)}\right)}^{\omega } \end{array} \end{array}\right)$$

$$\left(\begin{array}{c} \begin{array}{c} \sqrt[{q}]{1-{\left(1-\sin\left(\frac{\pi }{2}\mu_{\;\underline{Q1}}\right)\right)}^{\omega }},\\ {\left(\sqrt[{q}]{1-\sin^{q}\left(\frac{\pi }{2}\sqrt[{q}]{1-\zeta_{\;\underline{Q1}}^{q}}\;\right)}\right)}^{\omega }{\left(\sqrt[{q}]{1-\sin^{q}\left(\frac{\pi }{2}\sqrt[{q}]{1-\upsilon_{\underline{Q1}}^{q}}\right)}\right)}^{\omega } \end{array},\\ \begin{array}{c} \sqrt[{q}]{1-{\left(1-\sin\left(\frac{\pi }{2}\mu_{\;{\;\bar{Q}}_{1}}\right)\right)}^{\omega }},\\ {\left(\sqrt[{q}]{1-\sin^{q}\left(\frac{\pi }{2}\sqrt[{q}]{1-\zeta_{{\;\bar{Q}}_{1}}^{q}}\;\right)}\right)}^{\omega }{\left(\sqrt[{q}]{1-\sin^{q}\left(\frac{\pi }{2}\sqrt[{q}]{1-\upsilon_{{\;\bar{Q}}_{1}}^{q}}\right)}\right)}^{\omega } \end{array} \end{array}\right)⊕\left(\begin{array}{c} \begin{array}{c} \sqrt[{q}]{1-{\left(1-\sin\left(\frac{\pi }{2}\mu_{\;\underline{Q2}}\right)\right)}^{\omega }},\\ {\left(\sqrt[{q}]{1-\sin^{q}\left(\frac{\pi }{2}\sqrt[{q}]{1-\zeta_{\;\underline{Q2}}^{q}}\;\right)}\right)}^{\omega }{\left(\sqrt[{q}]{1-\sin^{q}\left(\frac{\pi }{2}\sqrt[{q}]{1-\upsilon_{\underline{Q2}}^{q}}\right)}\right)}^{\omega } \end{array},\\ \begin{array}{c} \sqrt[{p}]{1-{\left(1-\sin\left(\frac{\pi }{2}\mu_{\;{\;\bar{Q}}_{2}}\right)\right)}^{\omega }},\\ {\left(\sqrt[{q}]{1-\sin^{q}\left(\frac{\pi }{2}\sqrt[{q}]{1-\zeta_{\;{\;\bar{Q}}_{2}}^{q}}\;\right)}\right)}^{\omega }{\left(\sqrt[{q}]{1-\sin^{q}\left(\frac{\pi }{2}\sqrt[{q}]{1-\upsilon_{{\;\bar{Q}}_{2}}^{q}}\right)}\right)}^{\omega } \end{array} \end{array}\right)=\omega \;\sin\;{\;A}_{Q1\;}⊕\omega \;\sin\;{\;A}_{Q2\;}\text{.}$$

*Therefore*, *the first part of the proof has been concluded*.

*Now*, *for*$\omega_{1}$, $\omega_{2}≻0$, *we have*

$$\omega_{1}\;\sin\;{\;A}_{Q\;}=\left(\begin{array}{c} \begin{array}{c} \sqrt[{q}]{1-{\left(1-\sin\left(\frac{\pi }{2}\mu_{\;\underline{Q}}\right)\right)}^{\omega_{1}}},\\ {\left(\sqrt[{q}]{1-\sin^{q}\left(\frac{\pi }{2}\sqrt[{q}]{1-\zeta_{\;\underline{Q}}^{q}}\;\right)}\right)}^{\omega_{1}}{\left(\sqrt[{q}]{1-\sin^{q}\left(\frac{\pi }{2}\sqrt[{q}]{1-\upsilon_{\underline{Q}}^{q}}\right)}\right)}^{\omega_{1}} \end{array},\\ \begin{array}{c} \sqrt[{q}]{1-{\left(1-\sin\left(\frac{\pi }{2}\mu_{\;Q}\right)\right)}^{\omega_{1}}},\\ {\left(\sqrt[{q}]{1-\sin^{q}\left(\frac{\pi }{2}\sqrt[{q}]{1-\zeta_{\;\underline{Q}}^{q}}\;\right)}\right)}^{\omega_{1}}{\left(\sqrt[{q}]{1-\sin^{q}\left(\frac{\pi }{2}\sqrt[{q}]{1-\upsilon_{\underline{Q}}^{q}}\right)}\right)}^{\omega_{1}} \end{array} \end{array}\right)\text{and}$$

$$\omega_{2}\;\sin\;{\;A}_{Q\;}=\left(\begin{array}{c} \begin{array}{c} \sqrt[{q}]{1-{\left(1-\sin\left(\frac{\pi }{2}\mu_{\;\underline{Q}}\right)\right)}^{\omega_{2}}},\\ {\left(\sqrt[{q}]{1-\sin^{q}\left(\frac{\pi }{2}\sqrt[{q}]{1-\zeta_{\;\underline{Q}}^{q}}\;\right)}\right)}^{\omega_{2}}{\left(\sqrt[{q}]{1-\sin^{q}\left(\frac{\pi }{2}\sqrt[{q}]{1-\upsilon_{\underline{Q}}^{q}}\right)}\right)}^{\omega_{2}} \end{array},\\ \begin{array}{c} \sqrt[{q}]{1-{\left(1-\sin\left(\frac{\pi }{2}\mu_{\;\bar{Q}}\right)\right)}^{\omega_{2}}},\\ {\left(\sqrt[{q}]{1-\sin^{q}\left(\frac{\pi }{2}\sqrt[{q}]{1-\zeta_{\;\bar{Q}}^{q}}\;\right)}\right)}^{\omega_{2}}{\left(\sqrt[{q}]{1-\sin^{q}\left(\frac{\pi }{2}\sqrt[{q}]{1-\upsilon_{\bar{Q}}^{q}}\right)}\right)}^{\omega_{2}} \end{array} \end{array}\right)\text{,}$$

*By using the q-SFRNs' operating laws*, *we conclude at*

$$\omega_{1}\;\sin\;{\;A}_{Q}\omega_{2}\;\sin\;{\;A}_{Q\;}=\left(\begin{array}{c} \begin{array}{c} \sqrt[{q}]{1-{\left(1-\sin\left(\frac{\pi }{2}\mu_{\;\underline{Q}}\right)\right)}^{\omega_{1}}},\\ {\left(\sqrt[{q}]{1-\sin^{q}\left(\frac{\pi }{2}\sqrt[{q}]{1-\zeta_{\;\underline{Q}}^{q}}\;\right)}\right)}^{\omega_{1}}{\left(\sqrt[{q}]{1-\sin^{q}\left(\frac{\pi }{2}\sqrt[{q}]{1-\upsilon_{\underline{Q}}^{q}}\right)}\right)}^{\omega_{1}} \end{array},\\ \begin{array}{c} \sqrt[{q}]{1-{\left(1-\sin\left(\frac{\pi }{2}\mu_{\;\bar{Q}}\right)\right)}^{\omega_{1}}},\\ {\left(\sqrt[{q}]{1-\sin^{q}\left(\frac{\pi }{2}\sqrt[{q}]{1-\zeta_{\;\bar{Q}}^{q}}\;\right)}\right)}^{\omega_{1}}{\left(\sqrt[{q}]{1-\sin^{q}\left(\frac{\pi }{2}\sqrt[{q}]{1-\upsilon_{\bar{Q}}^{q}}\right)}\right)}^{\omega_{1}} \end{array} \end{array}\right)⊕$$

$$\left(\begin{array}{c} \begin{array}{c} \sqrt[{q}]{1-{\left(1-\sin\left(\frac{\pi }{2}\mu_{\;\underline{Q}}\right)\right)}^{\omega_{2}}},\\ {\left(\sqrt[{q}]{1-\sin^{q}\left(\frac{\pi }{2}\sqrt[{q}]{1-\zeta_{\;\underline{Q}}^{q}}\;\right)}\right)}^{\omega_{2}},{\left(\sqrt[{q}]{1-\sin^{q}\left(\frac{\pi }{2}\sqrt[{q}]{1-\upsilon_{\underline{Q}}^{q}}\right)}\right)}^{\omega_{2}} \end{array}\text{,}\\ \begin{array}{c} \sqrt[{q}]{1-{\left(1-\sin\left(\frac{\pi }{2}\mu_{\;\bar{Q}}\right)\right)}^{\omega_{2}}},\\ {\left(\sqrt[{q}]{1-\sin^{q}\left(\frac{\pi }{2}\sqrt[{q}]{1-\zeta_{\;\bar{Q}}^{q}}\;\right)}\right)}^{\omega_{2}},{\left(\sqrt[{q}]{1-\sin^{q}\left(\frac{\pi }{2}\sqrt[{q}]{1-\upsilon_{\bar{Q}}^{q}}\right)}\right)}^{\omega_{2}} \end{array} \end{array}\right)$$

$$=\left(\begin{array}{c} \begin{array}{c} \sqrt[{q}]{1-{\left(1-\sin\left(\frac{\pi }{2}\mu_{\;\underline{Q}}\right)\right)}^{\omega_{1}+\omega_{2}}},\\ {\left(\sqrt[{q}]{1-\sin^{q}\left(\frac{\pi }{2}\sqrt[{q}]{1-\zeta_{\;\underline{Q}}^{q}}\;\right)}\right)}^{\omega_{1}+\omega_{2}}{,\left(\sqrt[{q}]{1-\sin^{q}\left(\frac{\pi }{2}\sqrt[{q}]{1-\upsilon_{\underline{Q}}^{q}}\right)}\right)}^{\omega_{1}+\omega_{2}} \end{array},\\ \begin{array}{c} \sqrt[{p}]{1-{\left(1-\sin\left(\frac{\pi }{2}\mu_{\;\bar{Q}}\right)\right)}^{\omega_{1}+\omega_{2}}},\\ {\left(\sqrt[{q}]{1-\sin^{q}\left(\frac{\pi }{2}\sqrt[{q}]{1-\zeta_{\;\bar{Q}}^{q}}\;\right)}\right)}^{\omega_{1}+\omega_{2}}{,\left(\sqrt[{q}]{1-\sin^{q}\left(\frac{\pi }{2}\sqrt[{q}]{1-\upsilon_{\bar{Q}}^{q}}\right)}\right)}^{\omega_{1}+\omega_{2}} \end{array} \end{array}\right)=\left(\omega_{1}+\omega_{2}\right){\;A}_{Q}\text{.}$$

*As a result*, *the proof’s third segment has been finalized*.

Theorem 5 *For any two*q−SFRNs$\alpha =\left(\mu_{\underline{Q}},\zeta_{\underline{Q}},\upsilon_{\underline{Q}},\mu_{\;\bar{Q}},\zeta_{\;\bar{Q}},\upsilon_{\;\bar{Q}}\right)$*and*$\beta =\left(\mu_{\underline{R}},\zeta_{\underline{R}},\upsilon_{\underline{R}},\mu_{\;\bar{R}},\zeta_{\;\bar{R}},\upsilon_{\;\bar{R}}\right)$*we have*$\mu_{\underline{Q}}≽\mu_{\underline{R}},\mu_{\;\bar{Q}}≽\mu_{\;\bar{R}}$*which mean that*$\sin\left(\frac{\pi }{2}\mu_{\underline{Q}}\right)≽\sin\left(\frac{\pi }{2}\mu_{\underline{R}}\right)$*and*$\sin\left(\frac{\pi }{2}\mu_{\;\bar{Q}}\right)≽\sin\left(\frac{\pi }{2}\mu_{\;\bar{R}}\right)$ . *Similarly*, $\zeta_{\underline{Q}}≼\zeta_{\underline{R}},\zeta_{\;\bar{Q}}≼\zeta_{\;\bar{R}}$ *which implies that* $\sqrt[{q}]{1-{\zeta_{\underline{Q}}}^{q}}≽\sqrt[{q}]{1-{\zeta_{\underline{R}}}^{q}},\sqrt[{q}]{1-{\zeta_{\;\bar{Q}}}^{q}}≽\sqrt[{q}]{1-{\zeta_{\;\bar{R}}}^{q}},\sqrt[{q}]{1-{\upsilon_{\underline{Q}}}^{q}}≽\sqrt[{q}]{1-{\upsilon_{\underline{R}}}^{q}},\sqrt[{q}]{1-{\upsilon_{\;\bar{Q}}}^{q}}≽\sqrt[{q}]{1-{\upsilon_{\;\bar{R}}}^{q}}$. *Thus*, $\sin^{q}\left(\frac{\pi }{2}\sqrt[{q}]{1-{\upsilon_{\underline{Q}}}^{q}}\right)≼\sin^{q}\left(\frac{\pi }{2}\sqrt[{q}]{1-{\upsilon_{\underline{R}}}^{q}}\right),\sin^{q}\left(\frac{\pi }{2}\sqrt[{q}]{1-{\upsilon_{\;\bar{Q}}}^{q}}\right)≽\sin^{q}\left(\frac{\pi }{2}\sqrt[{q}]{1-{\upsilon_{\;\bar{R}}}^{q}}\right),\sin^{q}\left(\frac{\pi }{2}\sqrt[{q}]{1-{\zeta_{\underline{Q}}}^{q}}\right)≽\sin^{q}\left(\frac{\pi }{2}\sqrt[{q}]{1-{\zeta_{\underline{R}}}^{q}}\right),\sin^{q}\left(\frac{\pi }{2}\sqrt[{q}]{1-{\zeta_{\;\bar{Q}}}^{q}}\right)≽\sin^{q}\left(\frac{\pi }{2}\sqrt[{q}]{1-{\zeta_{\;\bar{R}}}^{q}}\right)$. *Therefore*, $\sqrt[{q}]{1-\sin^{q}\left(\frac{\pi }{2}\sqrt[{q}]{1-{\upsilon_{\underline{Q}}}^{q}}\right)}≼\sqrt[{q}]{1-\sin^{q}\left(\frac{\pi }{2}\sqrt[{q}]{1-{\zeta_{\underline{R}}}^{q}}\right)},\sqrt[{q}]{1-\sin^{q}\left(\frac{\pi }{2}\sqrt[{q}]{1-{\zeta_{\;\bar{Q}}}^{q}}\right)}≼\sqrt[{q}]{1-\sin^{q}\left(\frac{\pi }{2}\sqrt[{q}]{1-{\zeta_{\;\bar{R}}}^{q}}\right)},\sqrt[{q}]{1-{sin}^{q}\left(\frac{\pi }{2}\sqrt[{q}]{1-{\upsilon_{\underline{Q}}}^{q}}\right)}≼\sqrt[{q}]{1-\sin^{q}\left(\frac{\pi }{2}\sqrt[{q}]{1-{\upsilon_{\underline{R}}}^{q}}\right)},\sqrt[{q}]{1-\sin^{q}\left(\frac{\pi }{2}\sqrt[{q}]{1-{\upsilon_{\bar{Q}}}^{q}}\right)}≼\sqrt[{q}]{1-\sin^{q}\left(\frac{\pi }{2}\sqrt[{q}]{1-{\upsilon_{\;\bar{R}}}^{q}}\right)}$. *Hence*, *we have*

$$\left(\sin\left(\frac{\pi }{2}\mu_{\underline{Q}}\right),\sqrt[{q}]{1-\sin^{q}\left(\frac{\pi }{2}\sqrt[{q}]{1-\zeta_{\underline{Q}}^{q}}\right)},\sqrt[{q}]{1-\sin^{q}\left(\frac{\pi }{2}\sqrt[{q}]{1-\upsilon_{\underline{Q}}^{q}}\right)}\right)≽\left(\sin\left(\frac{\pi }{2}\mu_{\underline{R}}\right),\sqrt[{q}]{1-\sin^{q}\left(\frac{\pi }{2}\sqrt[{q}]{1-\zeta_{\underline{R}}^{q}}\right)},\sqrt[{q}]{1-\sin^{q}\left(\frac{\pi }{2}\sqrt[{q}]{1-\upsilon_{\underline{R}}^{q}}\right)}\right)\;and$$

$$\left(\sin\left(\frac{\pi }{2}\mu_{\;\bar{Q}}\right),\sqrt[{q}]{1-\sin^{q}\left(\frac{\pi }{2}\sqrt[{q}]{1-\zeta_{\;\bar{Q}}^{q}}\right)},\sqrt[{q}]{1-\sin^{q}\left(\frac{\pi }{2}\sqrt[{q}]{1-\upsilon_{\;\bar{Q}}^{q}}\right)}\right)≽\left(\sin\left(\frac{\pi }{2}\mu_{\bar{R}}\right),\sqrt[{q}]{1-\sin^{q}\left(\frac{\pi }{2}\sqrt[{q}]{1-\zeta_{\bar{R}}^{q}}\right)},\sqrt[{q}]{1-\sin^{q}\left(\frac{\pi }{2}\sqrt[{q}]{1-\upsilon_{\bar{R}}^{q}}\right)}\right)\text{.}$$

The proof is now complete.

Theorem 6 For any two q−SFRNs $\alpha_{j}=\left(\mu_{\underline{Q_{j}}},\zeta_{\underline{Q_{j}}},\upsilon_{\underline{Q_{j}}},\mu_{\;\bar{Q_{j}}},\zeta_{\;\bar{Q_{j}}},\upsilon_{\;\bar{Q_{j}}}\right)$ and $\alpha =\left(\mu_{\underline{Q}},\zeta_{\underline{Q}},\upsilon_{\underline{Q}},\mu_{\;\bar{Q}},\zeta_{\;\bar{Q}},\upsilon_{\;\bar{Q}}\right)$ we have $\sin\;\alpha_{j}⊕\sin\;\alpha ≽\sin\;\alpha_{j}⊗\sin\;\alpha$, where $\sin\;\alpha_{j}⊕\sin\;\alpha =\left(\sin\;{\underline{\alpha }}_{j}⊕\sin\underline{\alpha },\sin\;{\bar{\alpha }}_{j}⊕\sin\bar{\alpha }\right)$.

Proof For $\alpha_{j}$ and α. Take $\underline{x}=\sin^{p}\left(\frac{\pi }{2}\mu_{\underline{Q}}\right)$, ${\underline{x}}_{j}=\sin^{p}\left(\frac{\pi }{2}\mu_{\underline{Q_{j}}}\right)$, $\underline{y}=\sin^{q}\left(\frac{\pi }{2}\sqrt[{q}]{1-\zeta_{\;\bar{Q}}^{q}}\right)$, and ${\underline{y}}_{j}=\sin^{q}\left(\frac{\pi }{2}\sqrt[{q}]{1-\zeta_{{\underline{Q}}_{j}}^{q}}\right)$, and $\underline{z}=\sin^{q}\left(\frac{\pi }{2}\sqrt[{q}]{1-\upsilon_{\;\bar{Q}}^{q}}\right)$, and $z_{j}=\sin^{q}\left(\frac{\pi }{2}\sqrt[{q}]{1-\upsilon_{{\underline{Q}}_{j}}^{q}}\right)$ we have

(7) $$\sin\;{\underline{\alpha }}_{j}⊕\sin\underline{\alpha }=\left(\sqrt[{q}]{1-\left(1-\underline{x}\right)\left(1-{\underline{x}}_{j}\right)},\sqrt[{q}]{1-\underline{y}}\times \sqrt[{q}]{1-{\underline{y}}_{j}},\sqrt[{q}]{1-\underline{z}}\times \sqrt[{q}]{1-{\underline{z}}_{j}},\right)$$

and

(8) $$\sin\;{\underline{\alpha }}_{j}⊕\sin\underline{\alpha }=\left(\sqrt[{q}]{{\underline{x}}_{j}\times \underline{x}},\sqrt[{q}]{1-{\underline{y}}_{j}\times \underline{y}},\sqrt[{q}]{1-{\underline{z}}_{j}\times \underline{z}}\right)$$

Equation (7) and Equation (8) represents the lowe and upper sum of a sine trigonometric q-spherical fuzzy rough number.

Since $\underline{x}$, ${\underline{x}}_{j}$ and $\underline{y}$, ${\underline{y}}_{j}$ ∈[0,1], thus we have $\frac{\underline{x}+{\underline{x}}_{j}}{2}≽\underline{y}\times {\underline{y}}_{j}$ which implies that

$1-\left(1-\underline{x}\right)\left(1-\underline{x}\right)≽\underline{x}\times {\underline{x}}_{j}$, and thus $\sqrt[{q}]{1-\left(1-\underline{x}\right)\left(1-\underline{x}\right)≽}\sqrt[{q}]{\underline{x}\times {\underline{x}}_{j}}$, i.e.,

$$\sqrt[{q}]{1-\left(1-\sin^{p}\left(\frac{\pi }{2}\mu_{\underline{Q}}\right)\right)\times \left(1-\sin^{p}\left(\frac{\pi }{2}\mu_{{\underline{Q}}_{j}}\right)\right)≽\sqrt[{q}]{\sin^{p}\left(\frac{\pi }{2}\mu_{\underline{Q}}\right)\times \sin^{p}\left(\frac{\pi }{2}\mu_{{\underline{Q}}_{j}}\right)}}\text{.}$$

*Similarly*, $\sqrt[{q}]{1-\underline{y}}\times \sqrt[{q}]{1-{\underline{y}}_{j}}≼\sqrt[{q}]{1-\underline{y}\times {\underline{y}}_{j}}$*i*.*e*.,

$$\sqrt[{q}]{1-\sin^{q}\left(\frac{\pi }{2}\sqrt[{q}]{1-\zeta_{\underline{Q}}^{q}}\right)}\times \sqrt[{q}]{1-\sin^{q}\left(\frac{\pi }{2}\sqrt[{q}]{1-\zeta_{{\underline{Q}}_{j}}^{q}}\right)}$$

$$≼\sqrt[{q}]{1-1-\sin^{q}\left(\frac{\pi }{2}\sqrt[{q}]{1-\zeta_{\underline{Q}}^{q}}\right)\times 1-\sin^{q}\left(\frac{\pi }{2}\sqrt[{q}]{1-\zeta_{{\underline{Q}}_{j}}^{q}}\right)}\text{and}$$

$$\sqrt[{q}]{1-\sin^{q}\left(\frac{\pi }{2}\sqrt[{q}]{1-{\upsilon_{\underline{Q}}}^{q}}\right)}\times \sqrt[{q}]{1-\sin^{q}\left(\frac{\pi }{2}\sqrt[{q}]{1-\upsilon_{{\underline{Q}}_{j}}^{q}}\right)}$$

$$≼\sqrt[{q}]{1-1-\sin^{q}\left(\frac{\pi }{2}\sqrt[{q}]{1-\upsilon_{\underline{Q}}^{q}}\right)\times 1-\sin^{q}\left(\frac{\pi }{2}\sqrt[{q}]{1-\upsilon_{{\underline{Q}}_{j}}^{q}}\right)}\text{.}$$

We get the desired result because of Definition 3. Similarly, we can establish the same results for $\sin\;{\bar{\alpha }}_{j}⊕\sin\bar{\alpha }\text{.}$:

Theorem 7 For any positive real numbers ω and a q-SFRN $\alpha =\left(\mu_{\underline{Q}},\zeta_{\underline{Q}},\upsilon_{\underline{Q}},\mu_{\;\bar{Q}},\zeta_{\;\bar{Q}},\upsilon_{\;\bar{Q}}\right)$, the condition $\omega \;\sin\;\alpha ≽{\left(\sin\;\alpha \right)}^{\omega }$ if and only if 0 ≺ω≼1.

Proof The proof follows a similar method to that employed in Theorem 6.

## 4 Aggregations based on ST-q-SFRNs

Suppose that Δ represents a family of q-SFRNs $\alpha_{i}=\left(\mu_{{\underline{Q}}_{i}},\zeta_{{\underline{Q}}_{i}},\upsilon_{{\underline{Q}}_{i}},\mu_{{\;\bar{Q}}_{i}},\zeta_{{\;\bar{Q}}_{i}},\upsilon_{{\;\bar{Q}}_{i}}\right)$ and $\omega_{j}$ be the weight vector with the condition that $0≼\omega_{j}≼1$ and $\sum_{j=1}^{n}\omega_{j}=1\text{.}$ The requirement that the sum of the components of a weight vector equals 1 is a fundamental principle in decision-making and optimization, particularly in the context of multi-criteria decision-making (MCDM). This constraint is commonly known as normalization, and its purpose is to ensure that the weights assigned to various criteria are proportional and comparable, facilitating a meaningful and unbiased aggregation of criteria. When decision-makers assign weights to different criteria, these weights reflect the relative importance or contribution of each criterion to the overall decision. By normalizing the weights to sum up to 1, decision-makers are essentially expressing their preferences proportionally. The weight assigned to each criterion can be interpreted as the proportion of influence that criterion holds in the decision process. Normalization ensures that the weights are on a consistent scale, allowing for fair comparisons. Without normalization, decision-makers might assign weights on different scales, making it challenging to compare the impact of different criteria. A normalized weight vector provides a standardized measure, facilitating a more straightforward interpretation of the decision-makers' preferences. From a mathematical perspective, normalizing the weight vector simplifies the subsequent calculations involved in aggregating criteria. It ensures that the weights can be directly used as coefficients in a weighted sum or product, streamlining the decision-making process and enhancing the transparency of the methodology. Normalization helps avoid biases that may arise if decision-makers use different scales or units when assigning weights. By requiring the weights to sum up to 1, the decision-makers are compelled to distribute their preferences in a way that reflects a consistent and comprehensive assessment of criteria importance. A weight vector where the sum of the component to 1 provides a clear and interpretable representation of the decision-makers' preferences. Each weight can be easily understood as the proportion of the contribution of the corresponding criterion to the overall decision. We then express various operators using STSFRNs as follows.

Definition 9 A collection of "N" q-SFRNs is formed by $\alpha_{i}=\left(\mu_{{\underline{Q}}_{i}},\zeta_{{\underline{Q}}_{i}},\upsilon_{{\underline{Q}}_{i}},\mu_{{\;\bar{Q}}_{i}},\zeta_{{\;\bar{Q}}_{i}},\upsilon_{{\;\bar{Q}}_{i}}\right)$ (i=1,2,…,n). A mapping designated as ST-q-SFRWA: $\Delta^{n}\to \Delta$ is a sine trigonometric q-spherical fuzzy rough weighted averaging operator. Its definition is as follows:

(9) $$ST-q-SFRWA\left(\alpha_{1},\alpha_{2},\ldots ,\alpha_{n}\right)=\omega_{1}\;\sin\;\alpha_{1}⊕\omega_{2}\;\sin\;\alpha_{2}⊕\ldots ⊕\omega_{n}\;\sin\;\alpha_{n}$$

Equation (9) represents the sine trigonometric q-spherical fuzzy rough weighted averaging operator.

Theorem 8 *The aggregated values obtained by*ST−q−SFRWA*operator are still*q*-SFRN and get*

(10) $$ST-q-SFRWA\left(\alpha_{1},\alpha_{2},\ldots ,\alpha_{n}\right)=\left\langle \begin{array}{l} \sqrt[{q}]{1-\prod_{i=1}^{n}{\left(1-\sin^{p}\left(\frac{\pi }{2}\mu_{{\underline{Q}}_{i}}\right)\right)}^{\omega_{i}}},\prod_{i=1}^{n}{\left(\sqrt[{q}]{1-\sin^{q}\left(\frac{\pi }{2}\sqrt[{q}]{1-\zeta_{{\underline{Q}}_{i}}^{q}}\right),}\right)}^{\omega_{i}},\prod_{i=1}^{n}{\left(\sqrt[{q}]{1-\sin^{q}\left(\frac{\pi }{2}\sqrt[{q}]{1-\upsilon_{{\underline{Q}}_{i}}^{q}}\right),}\right)}^{\omega_{i}},\\ \sqrt[{q}]{1-\prod_{i=1}^{n}{\left(1-\sin^{p}\left(\frac{\pi }{2}\mu_{{\;\bar{Q}}_{i}}\right)\right)}^{\omega_{i}}},\prod_{i=1}^{n}{\left(\sqrt[{q}]{1-\sin^{q}\left(\frac{\pi }{2}\sqrt[{q}]{1-\zeta_{{\;\bar{Q}}_{i}}^{q}}\right),}\right)}^{\omega_{i}},\prod_{i=1}^{n}{\left(\sqrt[{q}]{1-\sin^{q}\left(\frac{\pi }{2}\sqrt[{q}]{1-\upsilon_{{\;\bar{Q}}_{i}}^{q}}\right),}\right)}^{\omega_{i}} \end{array}\right\rangle$$

Equation (10) represents that the aggregated values obtained by ST−q−SFRWA operator are still q-SFRN.

Proof We use a mathematical induction technique, starting with step 1, to prove the validity of Theorem 8.

**Sept 1.** When n=2, we can observe the following

$$ST-q-SFRWA\left(\alpha_{1},\alpha_{2}\right)=\omega_{1}\;\sin\;\alpha_{1}⊕\omega_{2}\;\sin\;\alpha_{2}\text{.}$$

By Definition 8, $\sin\;\alpha_{1}=\left\langle \begin{array}{l} \sin\left(\frac{\pi }{2}\mu_{{\underline{Q}}_{1}}\right),\sqrt[{q}]{1-\sin^{q}\left(\frac{\pi }{2}\sqrt[{q}]{1-\zeta_{{\underline{Q}}_{1}}^{q}}\right)},\sqrt[{q}]{1-\sin^{q}\left(\frac{\pi }{2}\sqrt[{q}]{1-\upsilon_{{\underline{Q}}_{1}}^{q}}\right),}\\ \sin\left(\frac{\pi }{2}\mu_{{\bar{Q}}_{1}}\right),\sqrt[{q}]{1-\sin^{q}\left(\frac{\pi }{2}\sqrt[{q}]{1-\zeta_{{\bar{Q}}_{1}}^{q}}\right)},\sqrt[{q}]{1-\sin^{q}\left(\frac{\pi }{2}\sqrt[{q}]{1-\upsilon_{{\bar{Q}}_{1}}^{q}}\right)} \end{array}\right\rangle$ and $\sin\;\alpha_{2}=\left\langle \begin{array}{l} \sin\left(\frac{\pi }{2}\mu_{{\underline{Q}}_{1}}\right),\sqrt[{q}]{1-\sin^{q}\left(\frac{\pi }{2}\sqrt[{q}]{1-\zeta_{{\underline{Q}}_{1}}^{q}}\right)},\sqrt[{q}]{1-\sin^{q}\left(\frac{\pi }{2}\sqrt[{q}]{1-\upsilon_{{\underline{Q}}_{1}}^{q}}\right),}\\ \sin\left(\frac{\pi }{2}\mu_{{\bar{Q}}_{1}}\right),\sqrt[{q}]{1-\sin^{q}\left(\frac{\pi }{2}\sqrt[{q}]{1-\zeta_{{\bar{Q}}_{1}}^{q}}\right)},\sqrt[{q}]{1-\sin^{q}\left(\frac{\pi }{2}\sqrt[{q}]{1-\upsilon_{{\bar{Q}}_{1}}^{q}}\right)} \end{array}\right\rangle \text{.}$

$$\omega_{1}\;\sin\;\alpha_{1}⊕\omega_{2}\;\sin\;\alpha_{2}=$$

$$\left\langle \begin{array}{l} \sqrt[{q}]{1-{\left(1-\sin^{p}\left(\frac{\pi }{2}\mu_{{\underline{Q}}_{1}}\right)\right)}^{\omega_{1}}},{\left(\sqrt[{q}]{1-\sin^{q}\left(\frac{\pi }{2}\sqrt[{q}]{1-\zeta_{{\underline{Q}}_{1}}^{q}}\right)}\right)}^{\omega_{1}},{\left(\sqrt[{q}]{1-\sin^{q}\left(\frac{\pi }{2}\sqrt[{q}]{1-\upsilon_{{\underline{Q}}_{1}}^{q}}\right)}\right)}^{\omega_{1}},\\ \sqrt[{q}]{1-{\left(1-\sin^{p}\left(\frac{\pi }{2}\mu_{{\bar{Q}}_{1}}\right)\right)}^{\omega_{1}}},{\left(\sqrt[{q}]{1-\sin^{q}\left(\frac{\pi }{2}\sqrt[{q}]{1-\zeta_{{\bar{Q}}_{1}}^{q}}\right)}\right)}^{\omega_{1}},{\left(\sqrt[{q}]{1-\sin^{q}\left(\frac{\pi }{2}\sqrt[{q}]{1-\upsilon_{{\bar{Q}}_{1}}^{q}}\right)}\right)}^{\omega_{1}} \end{array}\right\rangle ⊕\left\langle \begin{array}{l} \sqrt[{q}]{1-{\left(1-\sin^{p}\left(\frac{\pi }{2}\mu_{{\underline{Q}}_{2}}\right)\right)}^{\omega_{1}}},{\left(\sqrt[{q}]{1-\sin^{q}\left(\frac{\pi }{2}\sqrt[{q}]{1-\zeta_{{\underline{Q}}_{2}}^{q}}\right)}\right)}^{\omega_{1}},{\left(\sqrt[{q}]{1-\sin^{q}\left(\frac{\pi }{2}\sqrt[{q}]{1-\upsilon_{{\underline{Q}}_{2}}^{q}}\right)}\right)}^{\omega_{1}},\\ \sqrt[{q}]{1-{\left(1-\sin^{p}\left(\frac{\pi }{2}\mu_{{\bar{Q}}_{2}}\right)\right)}^{\omega_{1}}},{\left(\sqrt[{q}]{1-\sin^{q}\left(\frac{\pi }{2}\sqrt[{q}]{1-\zeta_{{\bar{Q}}_{2}}^{q}}\right)}\right)}^{\omega_{1}},{\left(\sqrt[{q}]{1-\sin^{q}\left(\frac{\pi }{2}\sqrt[{q}]{1-\upsilon_{{\bar{Q}}_{2}}^{q}}\right)}\right)}^{\omega_{1}} \end{array}\right\rangle$$

$$=\left\langle \begin{array}{l} \sqrt[{q}]{1-\prod_{i=1}^{2}{\left(1-\sin^{p}\left(\frac{\pi }{2}\mu_{{\underline{Q}}_{i}}\right)\right)}^{\omega_{i}}},\prod_{i=1}^{2}{\left(\sqrt[{q}]{1-\sin^{q}\left(\frac{\pi }{2}\sqrt[{q}]{1-\zeta_{{\underline{Q}}_{i}}^{q}}\right)}\right)}^{\omega_{i}},\prod_{i=1}^{2}{\left(\sqrt[{q}]{1-\sin^{q}\left(\frac{\pi }{2}\sqrt[{q}]{1-\upsilon_{{\underline{Q}}_{i}}^{q}}\right)}\right)}^{\omega_{i}}\\ \sqrt[{q}]{1-\prod_{i=1}^{2}{\left(1-\sin^{p}\left(\frac{\pi }{2}\mu_{{\bar{Q}}_{i}}\right)\right)}^{\omega_{i}}},\prod_{i=1}^{2}{\left(\sqrt[{q}]{1-\sin^{q}\left(\frac{\pi }{2}\sqrt[{q}]{1-\zeta_{{\bar{Q}}_{i}}^{q}}\right)}\right)}^{\omega_{i}},\prod_{i=1}^{2}{\left(\sqrt[{q}]{1-\sin^{q}\left(\frac{\pi }{2}\sqrt[{q}]{1-\upsilon_{{\bar{Q}}_{i}}^{q}}\right)}\right)}^{\omega_{i}} \end{array}\right\rangle \text{.}$$

Thus, Equation (10) is valid for n=2.

**Step 2.** We assume that Equation (10) holds for n=k, meaning that

$$ST-q-SFRFWA\left(\alpha_{1},\alpha_{2},\ldots ,\alpha_{k}\right)=\omega_{1}\;\sin\;\alpha_{1}⊕\omega_{2}\;\sin\;\alpha_{2}⊕\ldots ⊕\omega_{k}\alpha_{k}$$

$$=\left\langle \begin{array}{l} \sqrt[{q}]{1-\prod_{i=1}^{k}{\left(1-\sin^{p}\left(\frac{\pi }{2}\mu_{{\underline{Q}}_{i}}\right)\right)}^{\omega_{i}}},\prod_{i=1}^{k}{\left(\sqrt[{q}]{1-\sin^{q}\left(\frac{\pi }{2}\sqrt[{q}]{1-\zeta_{{\underline{Q}}_{i}}^{q}}\right)}\right)}^{\omega_{i}},\prod_{i=1}^{k}{\left(\sqrt[{q}]{1-\sin^{q}\left(\frac{\pi }{2}\sqrt[{q}]{1-\upsilon_{{\underline{Q}}_{i}}^{q}}\right)}\right)}^{\omega_{i}},\\ \sqrt[{q}]{1-\prod_{i=1}^{k}{\left(1-\sin^{p}\left(\frac{\pi }{2}\mu_{{\bar{Q}}_{i}}\right)\right)}^{\omega_{i}}},\prod_{i=1}^{k}{\left(\sqrt[{q}]{1-\sin^{q}\left(\frac{\pi }{2}\sqrt[{q}]{1-\zeta_{{\bar{Q}}_{i}}^{q}}\right)}\right)}^{\omega_{i}},\prod_{i=1}^{k}{\left(\sqrt[{q}]{1-\sin^{q}\left(\frac{\pi }{2}\sqrt[{q}]{1-\upsilon_{{\bar{Q}}_{i}}^{q}}\right)}\right)}^{\omega_{i}} \end{array}\right\rangle \text{.}$$

**Step 3.** Using the supposition from the previous stage, we prove the validity of the claim for the next natural number, n=k+1. We prove the correctness of the claim for the next natural number, n=k+1, by leveraging the assumption from the previous step.

$$ST-q-QOFWA\left(\alpha_{1},\alpha_{2},\ldots ,\alpha_{k+1}\right)=\omega_{1}\;\sin\;\alpha_{1}⊕\omega_{2}\;\sin\;\alpha_{2}⊕\ldots ⊕\omega_{k+1}\alpha_{k+1}$$

$$=\left\langle \begin{array}{l} \sqrt[{q}]{1-\prod_{i=1}^{k}{\left(1-\sin^{p}\left(\frac{\pi }{2}\mu_{{\underline{Q}}_{i}}\right)\right)}^{\omega_{i}}},\prod_{i=1}^{k}{\left(\sqrt[{q}]{1-\sin^{q}\left(\frac{\pi }{2}\sqrt[{q}]{1-\zeta_{{\underline{Q}}_{i}}^{q}}\right)}\right)}^{\omega_{i}}\prod_{i=1}^{k}{\left(\sqrt[{q}]{1-\sin^{q}\left(\frac{\pi }{2}\sqrt[{q}]{1-\upsilon_{{\underline{Q}}_{i}}^{q}}\right)}\right)}^{\omega_{i}},\\ \sqrt[{q}]{1-\prod_{i=1}^{k}{\left(1-\sin^{p}\left(\frac{\pi }{2}\mu_{{\bar{Q}}_{i}}\right)\right)}^{\omega_{i}}},\prod_{i=1}^{k}{\left(\sqrt[{q}]{1-\sin^{q}\left(\frac{\pi }{2}\sqrt[{q}]{1-\zeta_{{\bar{Q}}_{i}}^{q}}\right)}\right)}^{\omega_{i}}\prod_{i=1}^{k}{\left(\sqrt[{q}]{1-\sin^{q}\left(\frac{\pi }{2}\sqrt[{q}]{1-\upsilon_{{\bar{Q}}_{i}}^{q}}\right)}\right)}^{\omega_{i}} \end{array}\right\rangle ⊕\left\langle \begin{array}{l} \sqrt[{q}]{1-{\left(1-\sin^{p}\left(\frac{\pi }{2}\mu_{{\underline{Q}}_{k+1}}\right)\right)}^{\omega_{k+1}}},{\left(\sqrt[{q}]{1-\sin^{q}\left(\frac{\pi }{2}\sqrt[{q}]{1-\zeta_{{\underline{Q}}_{k+1}}^{q}}\right)}\right)}^{\omega_{k+1}},{\left(\sqrt[{q}]{1-\sin^{q}\left(\frac{\pi }{2}\sqrt[{q}]{1-\upsilon_{{\underline{Q}}_{k+1}}^{q}}\right)}\right)}^{\omega_{k+1}}\\ \sqrt[{q}]{1-{\left(1-\sin^{p}\left(\frac{\pi }{2}\mu_{{\bar{Q}}_{k+1}}\right)\right)}^{\omega_{k+1}}},{\left(\sqrt[{q}]{1-\sin^{q}\left(\frac{\pi }{2}\sqrt[{q}]{1-\zeta_{{\bar{Q}}_{k+1}}^{q}}\right)}\right)}^{\omega_{k+1}},{\left(\sqrt[{q}]{1-\sin^{q}\left(\frac{\pi }{2}\sqrt[{q}]{1-\upsilon_{{\bar{Q}}_{k+1}}^{q}}\right)}\right)}^{\omega_{k+1}} \end{array}\right\rangle$$

$$=\left\langle \begin{array}{l} \sqrt[{q}]{1-\prod_{i=1}^{k+1}{\left(1-\sin^{p}\left(\frac{\pi }{2}\mu_{{\underline{Q}}_{i}}\right)\right)}^{\omega_{i}}},\prod_{i=1}^{k+1}{\left(\sqrt[{q}]{1-\sin^{q}\left(\frac{\pi }{2}\sqrt[{q}]{1-\zeta_{{\underline{Q}}_{i}}^{q}}\right)}\right)}^{\omega_{i}},\prod_{i=1}^{k+1}{\left(\sqrt[{q}]{1-\sin^{q}\left(\frac{\pi }{2}\sqrt[{q}]{1-\upsilon_{{\underline{Q}}_{i}}^{q}}\right)}\right)}^{\omega_{i}},\\ \sqrt[{q}]{1-\prod_{i=1}^{k+1}{\left(1-\sin^{p}\left(\frac{\pi }{2}\mu_{{\bar{Q}}_{i}}\right)\right)}^{\omega_{i}}},\prod_{i=1}^{k+1}{\left(\sqrt[{q}]{1-\sin^{q}\left(\frac{\pi }{2}\sqrt[{q}]{1-\zeta_{{\bar{Q}}_{i}}^{q}}\right)}\right)}^{\omega_{i}},\prod_{i=1}^{k+1}{\left(\sqrt[{q}]{1-\sin^{q}\left(\frac{\pi }{2}\sqrt[{q}]{1-\upsilon_{{\bar{Q}}_{i}}^{q}}\right)}\right)}^{\omega_{i}} \end{array}\right\rangle \text{.}$$

According to the principle of mathematical induction, Equation (10) holds for all n.

Property 1 If all q-SFRNs $\alpha_{i}=\alpha$ then $\text{ST}-q-\text{SFRWA}\left(\alpha_{1},\alpha_{2},\ldots ,\alpha_{n}\right)=\sin\;\alpha$.

Property 2 Let $\alpha_{i}=\left(\mu_{{\underline{Q}}_{i}},\zeta_{{\underline{Q}}_{i}},\upsilon_{{\underline{Q}}_{i}},\mu_{{\;\bar{Q}}_{i}},\zeta_{{\;\bar{Q}}_{i}},\upsilon_{{\;\bar{Q}}_{i}}\right)$ (i=1,2,3,…,n) be the collection of “n” q-SFRNs. For

$\alpha^{-}=\left\langle \min\;\mu_{{\underline{Q}}_{i}},\max\;\zeta_{{\underline{Q}}_{i}},\max\;\upsilon_{{\underline{Q}}_{i}},\min\;\mu_{{\;\bar{Q}}_{i}},\max\;\zeta_{{\;\bar{Q}}_{i}},\max\;\upsilon_{{\bar{Q}}_{i}}\right\rangle$ and $\alpha^{+}=\left\langle \max\;\mu_{{\underline{Q}}_{i}},\min\;\zeta_{{\underline{Q}}_{i}},\min\;\upsilon_{{\underline{Q}}_{i}},\max\;\mu_{{\;\bar{Q}}_{i}},\min\;\zeta_{{\;\bar{Q}}_{i}},\min\;\upsilon_{{\bar{Q}}_{i}}\right\rangle$ then

$$\alpha^{-}≼ST-q-SFFRWA\left(\alpha_{1},\alpha_{2},\ldots ,\alpha_{n}\right)≼\alpha^{+}$$

Property 3 *For*q*-SFRNs*$\alpha_{i}=\left(\mu_{{\underline{Q}}_{i}},\zeta_{{\underline{Q}}_{i}},\upsilon_{{\underline{Q}}_{i}},\mu_{{\;\bar{Q}}_{i}},\zeta_{{\;\bar{Q}}_{i}},\upsilon_{{\;\bar{Q}}_{i}}\right)$*and*$\alpha_{i}^{*}=\left(\mu_{{\underline{Q}}_{i}}^{*},\zeta_{{\underline{Q}}_{i}}^{*},\upsilon_{{\underline{Q}}_{i}}^{*},\mu_{{\bar{Q}}_{i}}^{*},\zeta_{{\bar{Q}}_{i}}^{*},\upsilon_{{\bar{Q}}_{i}}^{*}\right)\text{.}$*If*$\mu_{{\underline{Q}}_{i}}≼\mu_{{\underline{Q}}_{i}}^{*}$, $\zeta_{{\underline{Q}}_{i}}≽\zeta_{{\underline{Q}}_{i}}^{*}$, $\upsilon_{{\underline{Q}}_{i}}≽\upsilon_{{\underline{Q}}_{i}}^{*}\;\text{and}\;\mu_{{\bar{Q}}_{i}}≼\mu_{{\bar{Q}}_{i}}^{*},\zeta_{{\bar{Q}}_{i}}≽\zeta_{{\bar{Q}}_{i}}^{*},\upsilon_{{\bar{Q}}_{i}}≽\upsilon_{{\bar{Q}}_{i}}^{*}$then

$$ST-q-SFRWA\left(\alpha_{1},\alpha_{2},\ldots ,\alpha_{n}\right)≼ST-q-SFRWA\left(\alpha_{i}^{*},\alpha_{i}^{*},\ldots ,\alpha_{i}^{*}\right)$$

Definition 10 *The collection of "n" q-SFRNs is represented as*$\alpha_{i}=\left(\mu_{{\underline{Q}}_{i}},\zeta_{{\underline{Q}}_{i}},\upsilon_{{\underline{Q}}_{i}},\mu_{{\;\bar{Q}}_{i}},\zeta_{{\;\bar{Q}}_{i}},\upsilon_{{\;\bar{Q}}_{i}}\right)$(i=1,2,3,…,n). *Then*, $ST-q-SFROWA:\Delta^{n}\to \Delta$*is a mapping that represents a sine trigonometric q-spherical fuzzy rough weighted averaging operator*. *It is defined by*

(11) $$ST-q-SFROWA\left(\alpha_{1},\alpha_{2},\ldots ,\alpha_{n}\right)=\omega_{1}\;\sin\;\alpha_{\delta \left(1\right)}⊕\omega_{2}\;\sin\;\alpha_{\delta \left(2\right)}⊕\ldots ⊕\omega_{n}\;\sin\;\alpha_{\delta \left(n\right)}$$

Equation (11) represent the sine trigonometric q-spherical fuzzy rough weighted averaging operator.

A key component is the permutation map δ, which needs to meet the requirement that, for i=1,2,3,…,n, $\alpha_{\delta \left(i\right)}$ is larger than or equal to $\alpha_{\delta \left(i-1\right)}$.

Theorem 9 The ST−q−SFROFWA operator yields aggregated values that are still q-SFRFN, resulting in

(12) $$ST-q-SFROWA\left(\alpha_{1},\alpha_{2},\ldots ,\alpha_{n}\right)=\left\langle \begin{array}{l} \sqrt[{q}]{1-\prod_{i=1}^{n}{\left(1-\sin^{p}\left(\frac{\pi }{2}\mu_{{\underline{Q}}_{\delta \left(i\right)}}\right)\right)}^{\omega_{i}}},\prod_{i=1}^{n}{\left(\sqrt[{q}]{1-\sin^{q}\left(\frac{\pi }{2}\sqrt[{q}]{1-\zeta_{{\underline{Q}}_{\delta \left(i\right)}}^{q}}\right)}\right)}^{\omega_{i}},\prod_{i=1}^{n}{\left(\sqrt[{q}]{1-\sin^{q}\left(\frac{\pi }{2}\sqrt[{q}]{1-\upsilon_{{\underline{Q}}_{\delta \left(i\right)}}^{q}}\right)}\right)}^{\omega_{i}},\\ \sqrt[{q}]{1-\prod_{i=1}^{n}{\left(1-\sin^{p}\left(\frac{\pi }{2}\mu_{{\bar{Q}}_{\delta \left(i\right)}}\right)\right)}^{\omega_{i}}},\prod_{i=1}^{n}{\left(\sqrt[{q}]{1-\sin^{q}\left(\frac{\pi }{2}\sqrt[{q}]{1-\zeta_{{\bar{Q}}_{\delta \left(i\right)}}^{q}}\right)}\right)}^{\omega_{i}},\prod_{i=1}^{n}{\left(\sqrt[{q}]{1-\sin^{q}\left(\frac{\pi }{2}\sqrt[{q}]{1-\upsilon_{{\bar{Q}}_{\delta \left(i\right)}}^{q}}\right)}\right)}^{\omega_{i}} \end{array}\right\rangle$$

Equation (12) represents that ST−q−SFROFWA operator yields aggregated values that are still q-SFRFN.

The proof follows the same steps as in Theorem 8.

Property 4 $\text{ST}-q-\text{QSFROWA}\left(\alpha_{1},\alpha_{2},\ldots ,\alpha_{n}\right)=\sin\;\alpha$ if all $\alpha_{\delta \left(i\right)}=\alpha$.

Property 5 Let the collection of "n" q-SFRNs be denoted by $\alpha_{i}=\left(\mu_{{\underline{Q}}_{i}},\zeta_{{\underline{Q}}_{i}},\upsilon_{{\underline{Q}}_{i}},\mu_{{\;\bar{Q}}_{i}},\zeta_{{\;\bar{Q}}_{i}},\upsilon_{{\;\bar{Q}}_{i}}\right)$ for all (i=1,2,…,n). For $\alpha^{-}=\left\langle \min\left\{\mu_{{\underline{Q}}_{\delta \left(i\right)}}\right\},\max\;\zeta_{{\underline{Q}}_{\delta \left(i\right)}},\max\;\upsilon_{{\underline{Q}}_{\delta \left(i\right)}},\min\;\mu_{{\bar{Q}}_{\delta \left(i\right)}},\max\;\zeta_{{\bar{Q}}_{\delta \left(i\right)}},\max\;\upsilon_{{\bar{Q}}_{\delta \left(i\right)}}\right\rangle$ and $\alpha^{+}=\left\langle \max\;\mu_{{\underline{Q}}_{\delta \left(i\right)}},\min\;\zeta_{{\underline{Q}}_{\delta \left(i\right)}},\min\;\upsilon_{{\underline{Q}}_{\delta \left(i\right)}},\max\;\mu_{{\bar{Q}}_{\delta \left(i\right)}},\min\;\zeta_{{\bar{Q}}_{\delta \left(i\right)}},\min\;\upsilon_{{\bar{Q}}_{\delta \left(i\right)}}\right\rangle$, then

$$\alpha^{-}≼ST-q-SFROWA\left(\alpha_{\delta \left(1\right)},\alpha_{\delta \left(2\right)},\ldots ,\alpha_{\delta \left(n\right)}\right)≼\alpha^{+}$$

Property 6 *For*q*-SFRNs*$\alpha_{i}=\left(\mu_{{\underline{Q}}_{i}},\zeta_{{\underline{Q}}_{i}},\upsilon_{{\underline{Q}}_{i}},\mu_{{\;\bar{Q}}_{i}},\zeta_{{\;\bar{Q}}_{i}},\upsilon_{{\;\bar{Q}}_{i}}\right)$*and*$\alpha_{i}^{*}=\left(\mu_{{\underline{Q}}_{i}}^{*},\zeta_{{\underline{Q}}_{i}}^{*},\upsilon_{{\underline{Q}}_{i}}^{*},\mu_{{\bar{Q}}_{i}}^{*},\zeta_{{\bar{Q}}_{i}}^{*},\upsilon_{{\bar{Q}}_{i}}^{*}\right)\text{.}$*If*$\mu_{{\underline{Q}}_{i}}≼\mu_{{\underline{Q}}_{i}}^{*}$, $\zeta_{{\underline{Q}}_{i}}≽\zeta_{{\underline{Q}}_{i}}^{*}$, $\upsilon_{{\underline{Q}}_{i}}≽\upsilon_{{\underline{Q}}_{i}}^{*}\;\text{and}\;\mu_{{\bar{Q}}_{i}}≼\mu_{{\bar{Q}}_{i}}^{*},\zeta_{{\bar{Q}}_{i}}≽\zeta_{{\bar{Q}}_{i}}^{*},\upsilon_{{\bar{Q}}_{i}}≽\upsilon_{{\bar{Q}}_{i}}^{*}$then

$$ST-q-SFROWA\left(\alpha_{1},\alpha_{2},\ldots ,\alpha_{n}\right)≼ST-q-SFROWA\left(\alpha_{i}^{*},\alpha_{i}^{*},\ldots ,\alpha_{i}^{*}\right)\text{.}$$

Definition 11 *Assume that*$\alpha_{i}=\left(\mu_{{\underline{Q}}_{i}},\zeta_{{\underline{Q}}_{i}},\upsilon_{{\underline{Q}}_{i}},\mu_{{\;\bar{Q}}_{i}},\zeta_{{\;\bar{Q}}_{i}},\upsilon_{{\;\bar{Q}}_{i}}\right)$(i=1,2,…,n). *Next*, *a mapping*$ST-q-SFRWG:\Delta^{n}\to \Delta$*is a sine trigonometric q-spherical fuzzy rough ordered weighted geometric* (ST−q−SFRWG) *operator*, *which is defined as follows*:

(13) $$ST-q-SFRWG\left(\alpha_{1},\alpha_{2},\ldots ,\alpha_{n}\right)={\left(\sin\;\alpha_{1}\right)}^{\omega_{1}}⊗{\left(\sin\;\alpha_{2}\right)}^{\omega_{2}}⊕\ldots ⊕{\left(\sin\;\alpha_{n}\right)}^{\omega_{n}}$$

Equation (13) represents the sine trigonometric q-spherical fuzzy rough ordered weighted geometric (ST−q−SFRWG) operator.

Theorem 10 *The aggregated values obtained by*ST−q−SFRWG*operator are still*q*-SFRN and get*

(14) $$ST-q-SFROWG\left(\alpha_{1},\alpha_{2},\ldots ,\alpha_{n}\right)=\left\langle \begin{array}{l} \sqrt[{q}]{\prod_{i=1}^{n}{\left(\sin^{q}\left(\frac{\pi }{2}\mu_{{\underline{Q}}_{i}}\right)\right)}^{\omega_{i}}},\sqrt[{q}]{{1-\prod_{i=1}^{n}\sin^{q}\left(\frac{\pi }{2}\sqrt[{q}]{1-\zeta_{{\underline{Q}}_{i}}^{q}}\right)}^{\omega_{i}},}\sqrt[{q}]{{1-\prod_{i=1}^{n}\sin^{q}\left(\frac{\pi }{2}\sqrt[{q}]{1-\upsilon_{{\underline{Q}}_{i}}^{q}}\right)}^{\omega_{i}},}\\ \sqrt[{q}]{\prod_{i=1}^{n}{\left(\sin^{q}\left(\frac{\pi }{2}\mu_{{\bar{Q}}_{i}}\right)\right)}^{\omega_{i}}},\sqrt[{q}]{{1-\prod_{i=1}^{n}\sin^{q}\left(\frac{\pi }{2}\sqrt[{q}]{1-\zeta_{{\bar{Q}}_{i}}^{q}}\right)}^{\omega_{i}},}\sqrt[{q}]{{1-\prod_{i=1}^{n}\sin^{q}\left(\frac{\pi }{2}\sqrt[{q}]{1-\upsilon_{{\bar{Q}}_{i}}^{q}}\right)}^{\omega_{i}}} \end{array}\right\rangle$$

Equation (14) represents that the aggregated values obtained by ST−q−SFRWG operator are still q-SFRN.

The proof follows the same steps as in Theorem 8.

Property 7 $\text{ST}-q-\text{SFRWG}\left(\alpha_{1},\alpha_{2},\ldots ,\alpha_{n}\right)=\sin\;\alpha$, if all q-SFRNs have $\alpha_{i}=\alpha$

Property 8 Let the family of " n" q−SFRNs be represented by $\alpha_{i}=\left(\mu_{{\underline{Q}}_{i}},\zeta_{{\underline{Q}}_{i}},\upsilon_{{\underline{Q}}_{i}},\mu_{{\;\bar{Q}}_{i}},\zeta_{{\;\bar{Q}}_{i}},\upsilon_{{\;\bar{Q}}_{i}}\right)$ (i=1,2,…,n). In the case of

$\alpha^{-}=\left\langle \min\;\mu_{{\underline{Q}}_{i}},\max\;\zeta_{{\underline{Q}}_{i}},\max\;\upsilon_{{\underline{Q}}_{i}},\min\;\mu_{{\;\bar{Q}}_{i}},\max\;\zeta_{{\bar{Q}}_{i}},\max\;\upsilon_{{\bar{Q}}_{i}}\right\rangle$ and $\alpha^{+}=\left\langle \max\;\mu_{{\underline{Q}}_{i}},\min\;\zeta_{{\underline{Q}}_{i}},\min\;\upsilon_{{\underline{Q}}_{i}},\max\;\mu_{{\;\bar{Q}}_{i}},\min\;\zeta_{{\bar{Q}}_{i}},\min\;\upsilon_{{\bar{Q}}_{i}}\right\rangle \text{,}$ then

$$\alpha^{-}≼ST-q-SFRWG\left(\alpha_{1},\alpha_{2},\ldots ,\alpha_{n}\right)≼\alpha^{+}$$

Property 9 *For*q*-SFRNs*$\alpha_{i}=\left(\mu_{{\underline{Q}}_{i}},\zeta_{{\underline{Q}}_{i}},\upsilon_{{\underline{Q}}_{i}},\mu_{{\;\bar{Q}}_{i}},\zeta_{{\;\bar{Q}}_{i}},\upsilon_{{\;\bar{Q}}_{i}}\right)$*and*$\alpha_{i}^{*}=\left(\mu_{{\underline{Q}}_{i}}^{*},\zeta_{{\underline{Q}}_{i}}^{*},\upsilon_{{\underline{Q}}_{i}}^{*},\mu_{{\bar{Q}}_{i}}^{*},\zeta_{{\bar{Q}}_{i}}^{*},\upsilon_{{\bar{Q}}_{i}}^{*}\right)\text{.}$*If*$\mu_{{\underline{Q}}_{i}}≼\mu_{{\underline{Q}}_{i}}^{*}$, $\zeta_{{\underline{Q}}_{i}}≽\zeta_{{\underline{Q}}_{i}}^{*}$, $\upsilon_{{\underline{Q}}_{i}}≽\upsilon_{{\underline{Q}}_{i}}^{*}$and$\mu_{{\bar{Q}}_{i}}≼\mu_{{\bar{Q}}_{i}}^{*},\zeta_{{\bar{Q}}_{i}}≽\zeta_{{\bar{Q}}_{i}}^{*},\upsilon_{{\bar{Q}}_{i}}≽\upsilon_{{\bar{Q}}_{i}}^{*}$, *then*

$$ST-q-SFRWG\left(\alpha_{1},\alpha_{2},\ldots ,\alpha_{n}\right)≼ST-q-SFRWG\left(\alpha_{i}^{*},\alpha_{i}^{*},\ldots ,\alpha_{i}^{*}\right)\text{.}$$

Definition 12 *The family of*$\prime \prime n^{\prime \prime }\;q-SFRFNs$*is represented by*$\alpha_{i}=\left(\mu_{{\underline{Q}}_{i}},\zeta_{{\underline{Q}}_{i}},\upsilon_{{\underline{Q}}_{i}},\mu_{{\;\bar{Q}}_{i}},\zeta_{{\;\bar{Q}}_{i}},\upsilon_{{\;\bar{Q}}_{i}}\right)$(i=1,2,…,n). *The mapping*$ST-q-SFROWG:\Delta^{n}\to \Delta$*is the sine trigonometric q-spherical fuzzy rough ordered weighted geometric*(ST−q−SFROWG)*operators*, *which are defined as follows*:

(15) $$ST-q-SFROWG\left(\alpha_{1},\alpha_{2},\ldots ,\alpha_{n}\right)={\left(\sin\;\alpha_{1}\right)}^{\omega_{1}}⊗{\left(\sin\;\alpha_{2}\right)}^{\omega_{2}}⊕\ldots ⊕{\left(\sin\;\alpha_{n}\right)}^{\omega_{n}}$$

Equation (15) represents that the mapping $\text{ST}-q-\text{SFROWG}:\Delta^{n}\to \Delta$ is the sine trigonometric q-spherical fuzzy rough ordered weighted geometric (ST−q−SFROWG) operators.

Theorem 11 *The aggregated values obtained by*ST−q−SFROWG*operator are still*q*-SFRN and*

(16) $$ST-q-SFROWG\left(\alpha_{1},\alpha_{2},\ldots ,\alpha_{n}\right)=\left\langle \begin{array}{l} \sqrt[{q}]{\prod_{i=1}^{n}{\left(\sin^{p}\left(\frac{\pi }{2}\mu_{{\underline{Q}}_{i}}\right)\right)}^{\omega_{i}}},\sqrt[{q}]{{1-\prod_{i=1}^{n}\sin^{q}\left(\frac{\pi }{2}\sqrt[{q}]{1-\zeta_{{\underline{Q}}_{i}}^{q}}\right)}^{\omega_{i}}},\sqrt[{q}]{{1-\prod_{i=1}^{n}\sin^{q}\left(\frac{\pi }{2}\sqrt[{q}]{1-\upsilon_{{\underline{Q}}_{i}}^{q}}\right)}^{\omega_{i}}}\\ \sqrt[{q}]{\prod_{i=1}^{n}{\left(\sin^{p}\left(\frac{\pi }{2}\mu_{{\bar{Q}}_{i}}\right)\right)}^{\omega_{i}}},\sqrt[{q}]{{1-\prod_{i=1}^{n}\sin^{q}\left(\frac{\pi }{2}\sqrt[{q}]{1-\zeta_{{\bar{Q}}_{i}}^{q}}\right)}^{\omega_{i}}},\sqrt[{q}]{{1-\prod_{i=1}^{n}\sin^{q}\left(\frac{\pi }{2}\sqrt[{q}]{1-\upsilon_{{\bar{Q}}_{i}}^{q}}\right)}^{\omega_{i}}} \end{array},\right\rangle$$

Equation (16) represents that the aggregated values obtained by ST−q−SFROWG operator are still q-SFRN.

The proof follows the same steps as in Theorem 8.

Property 10 $\text{ST}-q-\text{SFROWG}\left(\alpha_{1},\alpha_{2},\ldots ,\alpha_{n}\right)=\sin\;\alpha$ if all q-SFRNs have $\alpha_{i}=\alpha$.

Property 11 Let a family of "n" q-SFRNs be represented as $\alpha_{i}=\left(\mu_{{\underline{Q}}_{i}},\zeta_{{\underline{Q}}_{i}},\upsilon_{{\underline{Q}}_{i}},\mu_{{\;\bar{Q}}_{i}},\zeta_{{\;\bar{Q}}_{i}},\upsilon_{{\;\bar{Q}}_{i}}\right)$ (i=1,2,…,n).

*For*$\alpha^{-}=\left\langle \min\;\mu_{{\underline{Q}}_{i}},\max\;\zeta_{{\underline{Q}}_{i}},\max\;\upsilon_{{\underline{Q}}_{i}},\min\;\mu_{{\;\bar{Q}}_{i}},\max\;\zeta_{{\bar{Q}}_{i}},\max\;\upsilon_{{\bar{Q}}_{i}}\right\rangle$*and*$\alpha^{+}=\left\langle \max\;\mu_{{\underline{Q}}_{i}},\min\;\zeta_{{\underline{Q}}_{i}},\min\;\upsilon_{{\underline{Q}}_{i}},\max\;\mu_{{\;\bar{Q}}_{i}},\min\;\zeta_{{\bar{Q}}_{i}},\min\;\upsilon_{{\bar{Q}}_{i}}\right\rangle \text{,}$*then*

$$\alpha^{-}≼ST-q-SFROWG\left(\alpha_{1},\alpha_{2},\ldots ,\alpha_{n}\right)≼\alpha^{+}$$

Property 12 *For*q*-SFRNs*$\alpha_{i}=\left(\mu_{{\underline{Q}}_{i}},\zeta_{{\underline{Q}}_{i}},\upsilon_{{\underline{Q}}_{i}},\mu_{{\;\bar{Q}}_{i}},\zeta_{{\;\bar{Q}}_{i}},\upsilon_{{\;\bar{Q}}_{i}}\right)$*and*$\alpha_{i}^{*}=\left(\mu_{{\underline{Q}}_{i}}^{*},\zeta_{{\underline{Q}}_{i}}^{*},\upsilon_{{\underline{Q}}_{i}}^{*},\mu_{{\bar{Q}}_{i}}^{*},\zeta_{{\bar{Q}}_{i}}^{*},\upsilon_{{\bar{Q}}_{i}}^{*}\right)\text{.}$*If*$\mu_{{\underline{Q}}_{i}}≼\mu_{{\underline{Q}}_{i}}^{*}$, $\zeta_{{\underline{Q}}_{i}}≽\zeta_{{\underline{Q}}_{i}}^{*}$, $\upsilon_{{\underline{Q}}_{i}}≽\upsilon_{{\underline{Q}}_{i}}^{*}\;\text{and}\;\mu_{{\bar{Q}}_{i}}≼\mu_{{\bar{Q}}_{i}}^{*},\zeta_{{\bar{Q}}_{i}}≽\zeta_{{\bar{Q}}_{i}}^{*},\upsilon_{{\bar{Q}}_{i}}≽\upsilon_{{\bar{Q}}_{i}}^{*}$then

$$ST-q-SFROWG\left(\alpha_{1},\alpha_{2},\ldots ,\alpha_{n}\right)≼ST-q-SFROWG\left(\alpha_{i}^{*},\alpha_{i}^{*},\ldots ,\alpha_{i}^{*}\right)\text{.}$$

## 5 Propose algorithm

Suppose that we have a decision-making problem with “m” alternatives labeled as $V_{1}$, $V_{2}$, …, $V_{m}$, and “n” attributes are denoted as $J_{1}$, $J_{2}$, …, $J_{n}$. The decision-making process involves “d” decision-makers. The decision-makers provide their input through standardized weight information, where $w_{k}≻0$ signifies the weight assigned by decision makers “d” and $\omega_{j}≻0$ represents the weight associated with the attribute $J_{j}$. In this case, " k" is between 1 and d, while " j” is between 0 and n. Using a framework based on q-SFRSs, every decision-maker evaluates each option $V_{i}$ about each attribute $J_{j}$. These assessments' outcomes are recorded and given as $\alpha_{ij}^{k}=\left(\mu_{{\underline{Q}}_{ij}}^{k},\zeta_{{\underline{Q}}_{ij}}^{k},\upsilon_{{\underline{Q}}_{ij}}^{k},\mu_{{\bar{Q}}_{ij}}^{k}\zeta_{{\bar{Q}}_{ij}}^{k},\upsilon_{{\bar{Q}}_{ij}}^{k}\right)$, where i, j, and " k " are the integers from 1 to m, 0 to n, and 1 to d, respectively. In conclusion, the following method is used to summarise each alternative $V_{i}$'s choice.

(17) $$V_{i}=\left\{\left(J_{1},\alpha_{i1}\right),\left(J_{2},\alpha_{i2}\right),\ldots ,\left(J_{n},\alpha_{in}\right)\right\}$$

Equation (17) represents that alternatives weight corresponding to associated attributes.

The suggested algorithm takes these steps to identify the best selection among the alternatives.

**Step 1.** For every decision maker, arrange and structure the matrices $D^{\left(k\right)}=\left(\alpha_{ij}^{\left(k\right)}\right)$. Each decision maker's recorded assessments are gathered and arranged during this phase. The assessments and inclinations of every decision-maker for every choice are documented in detail using these matrices.

**Step 2.** Gather the individual assessments $\left(\alpha_{ij}^{\left(k\right)}\right)$, where k is a number between 1 and d, and combine them into a single representation that is represented as $\alpha_{ij}^{k}=\left(\mu_{{\underline{Q}}_{ij}}^{k},\zeta_{{\underline{Q}}_{ij}}^{k},\upsilon_{{\underline{Q}}_{ij}}^{k},\mu_{{\bar{Q}}_{ij}}^{k}\zeta_{{\bar{Q}}_{ij}}^{k},\upsilon_{{\bar{Q}}_{ij}}^{k}\right)$. Any ST-q-SFROWG operator can be used to complete this aggregate. Following their combination, these values are organized into the matrix $D=\left(\alpha_{ij}\right)$. The resulting matrix provides a summary of the combined assessments and preferences for each alternative, which will be useful for making decisions.

**Step 3.** Standards are employed in the decision-making process to assess and compare the alternatives that are that are accessible. In this context, two sorts of criteria are used: benefit criteria and cost criteria. To choose the optimal choice for resolving the given problem, benefit criteria evaluate the positive aspects of the solutions under consideration, highlighting their advantages and benefits. Contrarily, cost criteria measure the drawbacks or expenses of all alternatives to evaluate their respective advantages. These economic factors are crucial in determining the viability of a solution. If necessary, the recorded data $\alpha_{ij}$ to $r_{ij}$ can be standardized by using the following rule:

(18) $$r_{ij}=\left\{ \begin{array}{c} \left(\mu_{{\underline{Q}}_{ij}}^{k},\zeta_{{\underline{Q}}_{ij}}^{k},\upsilon_{{\underline{Q}}_{ij}}^{k},\mu_{{\bar{Q}}_{ij}}^{k}\zeta_{{\bar{Q}}_{ij}}^{k},\upsilon_{{\bar{Q}}_{ij}}^{k}\right)\;\text{for}\;\text{benefit}\;\text{criteria}\\ \left(\upsilon_{{\underline{Q}}_{ij}}^{k},\mu_{{\underline{Q}}_{ij}}^{k},\zeta_{{\underline{Q}}_{ij}}^{k},\upsilon_{{\bar{Q}}_{ij}}^{k},\mu_{{\bar{Q}}_{ij}}^{k}\zeta_{{\bar{Q}}_{ij}}^{k}\right)\;\text{for}\;\text{cost}\;\text{criteria} \end{array}\right.$$

Equation (18) represents the formula for both the benefit type criteria and cost type criteria.

By standardizing the evaluations based on whether they are cost or benefit criteria, this phase facilitates decision-making by ensuring that the assessments are similar.

**Step 4.** Determine the entropy for each characteristic $J_{j}$ using Equation (19). The amount of uncertainty or variability connected to a certain attribute is measured by entropy. We can determine the degree of variance or consistency between the potential links between each attribute by calculating the entropy. This knowledge is useful in identifying the factors that influence decision-making the most and in identifying the alternatives that have the highest chance of yielding the desired results.

(19) $$E_{j}=\frac{1}{\left(\sqrt{2}-1\right)m}\sum_{i=1}^{m}\left(\sin\left(\frac{\pi }{4}\left(1+\left(\mu_{{\underline{Q}}_{ij}}^{q}+\mu_{{\bar{Q}}_{ij}}^{q}\right)-\left(\zeta_{{\underline{Q}}_{ij}}^{q}+\zeta_{{\bar{Q}}_{ij}}^{q}\right)-\left(\upsilon_{{\underline{Q}}_{ij}}^{q}+\upsilon_{{\bar{Q}}_{ij}}^{q}\right)\right)\right)+\sin\left(\frac{\pi }{4}\left(1-\left(\mu_{{\underline{Q}}_{ij}}^{q}+\mu_{{\bar{Q}}_{ij}}^{q}\right)-\left(\zeta_{{\underline{Q}}_{ij}}^{q}+\zeta_{{\bar{Q}}_{ij}}^{q}\right)-\left(\upsilon_{{\underline{Q}}_{ij}}^{q}+\upsilon_{{\bar{Q}}_{ij}}^{q}\right)\right)\right)\right)$$

Where $0≼E_{j}≼1$ is ensured by the constant $\frac{1}{\left(\sqrt{2}-1\right)m}$. If the attribute weights $\omega_{j}$ are unknown, find them using Equation (20).

In cases where the attribute weights $\omega_{j}$ are not known, you can derive them using Equation (20). This equation ensures that the weights fall within the range of 0–1. The constant term $\frac{1}{\left(\sqrt{2}-1\right)m}$ serves to normalize the entropy values, guaranteeing that they are bounded by 0–1. The attribute weights $\omega_{j}$ can be calculated by evaluating Equation (20) based on the calculated entropy values for each attribute, providing a way to assign relative importance to the attributes in the decision-making process.

(20) $$\omega_{j}=\frac{1-E_{j}}{n-\sum_{j=1}^{n}E_{j}}$$

**Step 5.** Utilize the recommended operator to merge the values of $r_{ij}$, covering the range of attributes from 1 to n, to derive the consolidated value $\alpha_{i}=\left(\mu_{{\underline{Q}}_{ij}}^{k},\zeta_{{\underline{Q}}_{ij}}^{k},\upsilon_{{\underline{Q}}_{ij}}^{k},\mu_{{\bar{Q}}_{ij}}^{k}\zeta_{{\bar{Q}}_{ij}}^{k},\upsilon_{{\bar{Q}}_{ij}}^{k}\right)$. In this step, the operator combines the standardized values of $r_{ij}$, from all attributes to produce a comprehensive representation, $\alpha_{i}$, when encapsulates to characteristics of the alternative under consideration.

**Step 6.** Calculate the score values for $r_{i}=\left(\mu_{{\underline{Q}}_{i}},\zeta_{{\underline{Q}}_{i}},\upsilon_{{\underline{Q}}_{i}},\mu_{{\;\bar{Q}}_{i}},\zeta_{{\;\bar{Q}}_{i}},\upsilon_{{\;\bar{Q}}_{i}}\right)$ by employing Equation (21).

(21) $$Sco\left(r_{i}\right)=\frac{1}{3}\left(2+\left(\mu_{{\underline{Q}}_{ij}}^{q}+\mu_{{\bar{Q}}_{ij}}^{q}\right)-\left(\zeta_{{\underline{Q}}_{ij}}^{q}+\zeta_{{\bar{Q}}_{ij}}^{q}\right)-\left(\upsilon_{{\underline{Q}}_{ij}}^{q}+\upsilon_{{\bar{Q}}_{ij}}^{q}\right)\right)$$

The variable q is a natural number.

**Step 7.** Arrange the various alternatives in $V_{i}$ according to how much you like each one, and then choose the alternative or alternatives that you think are best.

**Fig. 1:**
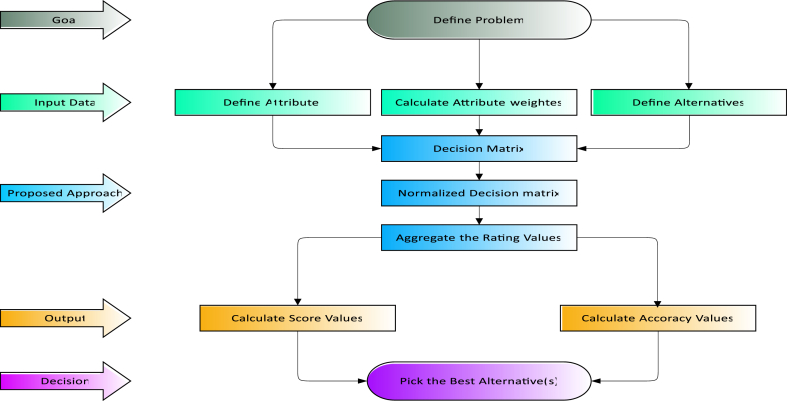
displays the typical framework of the multi-criteria decision-making approach.

Fig. 1 shows the multi-criteria decision-making (MCDM) process in a format that is commonly used. This structure, as shown in Fig. 1, typically consists of the following steps: defining criteria, giving these criterion weights, and adding up the scores to determine which selection is best.

### 5.1 Application

Example 1**.** To elucidate and illustrate the suggested technique, we offer an example in this section. In this scenario, a company is evaluating four technology vendors for their digital transformation initiative and the evaluation is based on four criteria "technology expertise", "cost", "customer support "and "innovative factors". A group of four experts will assign weights to these criteria, and we use a decision matrix and decision tree to make the selection.

**Scenario: Selecting a Digital Transformation Vendor**.

**Candidates (Vendors):**

$$V_{1}=\text{Tech}\;\text{Solution}\;\text{Inc.}$$

$$V_{2}=\text{Innovative}\;\text{Tech}\;\text{Corp.}$$

$$V_{3}=\text{Degital}\;\text{Tranform}\;\text{Ltd.}$$

$$V_{4}=\text{Future}\;\text{Tech}\;\text{Innovations}$$

**Criteria (Attributes):**

$$J_{1}=\text{Technolgy}\;\text{Expertise}$$

$$J_{2}=\text{Cost}$$

$$J_{3}=\text{Customer}\;\text{Support}$$

$$J_{4}=\text{Innovative}\;\text{factor}$$

**Weight Vector (Expert Opinion):**

w=(0.3,0.1,0.4,0.2).

**Fig. 2:**
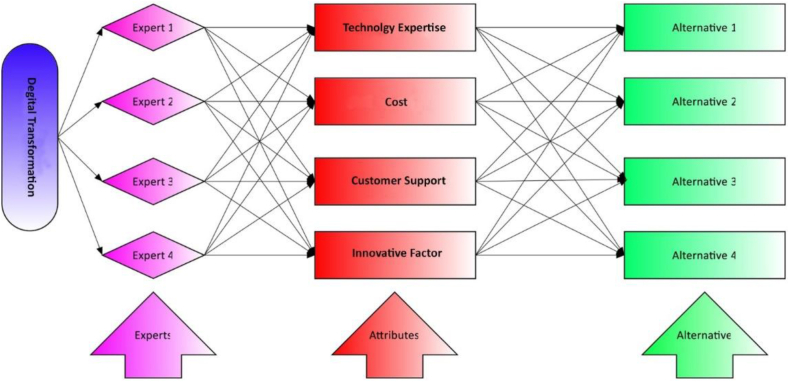
Decision tree for Digital transformation (Vendor).

The weight vector presents the importance of each criterion as determined by a group of experts. Now let's create a decision matrix $D^{\left(k\right)}=\left(\alpha_{ij}^{\left(k\right)}\right)$ for each vendor (k=1,2,3,4), Table 1, Table 2, Table 3, Table 4 provides the decision matrix for evaluating technology vendors. The goal is to rank these vendors and select the most suitable for the digital transformation project. Fig. 2. Illustrates a decision tree used for the vendor selection process in the context of technology procurement).

**Table 1:** Decision matrix $D^{1}$.

**Alternatives**	$J_{1}$	$J_{2}$	$J_{3}$	$J_{4}$
$V_{1}$	$\left(\begin{array}{c} 0.56,0.63,0.25\text{,}\\ 0.45,0.45,0.96 \end{array}\right)$	$\left(\begin{array}{c} 0.89,0.45,0.45\text{,}\\ 0.58,0.58,0.56 \end{array}\right)$	$\left(\begin{array}{c} 0.25,0.56,0.85\text{,}\\ 0.25,0.56,0.85 \end{array}\right)$	$\left(\begin{array}{c} 0.96,0.85,0.74\text{,}\\ 0.52,0.63,0.63 \end{array}\right)$
$V_{2}$	$\left(\begin{array}{c} 0.36,0.99,0.52\text{,}\\ 0.85,0.25,0.85 \end{array}\right)$	$\left(\begin{array}{c} 0.85,0.56,0.36\text{,}\\ 0.36,0.56,0.58 \end{array}\right)$	$\left(\begin{array}{c} 0.25,0.25,0.25\text{,}\\ 0.89,0.25,0.25 \end{array}\right)$	$\left(\begin{array}{c} 0.25,0.36,0.96\text{,}\\ 0.74,0.14,0.25 \end{array}\right)$
$V_{3}$	$\left(\begin{array}{c} 0.45,0.26,0.23\text{,}\\ 0.25,0.75,0.16 \end{array}\right)$	$\left(\begin{array}{c} 0.58,0.78,0.89\text{,}\\ 0.98,0.78,0.69 \end{array}\right)$	$\left(\begin{array}{c} 0.25,0.38,0.23\text{,}\\ 0.21,0.45,0.78 \end{array}\right)$	$\left(\begin{array}{c} 0.25,0.36,0.36\text{,}\\ 0.36,0.85,0.85 \end{array}\right)$
$V_{4}$	$\left(\begin{array}{c} 0.25,0.96,0.45\text{,}\\ 0.45,0.65,0.85 \end{array}\right)$	$\left(\begin{array}{c} 0.78,0.85,0.85\text{,}\\ 0.35,0.85,0.65 \end{array}\right)$	$\left(\begin{array}{c} 0.56,0.23,0.56\text{,}\\ 0.89,0.23,0.36 \end{array}\right)$	$\left(\begin{array}{c} 0.36,0.96,0.14\text{,}\\ 0.75,0.25,0.75 \end{array}\right)$

**Table 2:** Decision matrix $D^{2}$.

**Alternatives**	$J_{1}$	$J_{2}$	$J_{3}$	$J_{4}$
$V_{1}$	$\left(\begin{array}{c} 0.85,0.39,0.23\text{,}\\ 0.32,0.50,0.63 \end{array}\right)$	$\left(\begin{array}{c} 0.54,0.12,0.23\text{,}\\ 0.60,0.74,0.23 \end{array}\right)$	$\left(\begin{array}{c} 0.85,0.8,0.96\text{,}\\ 0.25,0.36,0.30 \end{array}\right)$	$\left(\begin{array}{c} 0.69,0.36,0.36\text{,}\\ 0.74,0.32,0.69 \end{array}\right)$
$V_{2}$	$\left(\begin{array}{c} 0.85,0.37,0.50\text{,}\\ 0.12,0.78,0.30 \end{array}\right)$	$\left(\begin{array}{c} 0.85,0.96,0.85\text{,}\\ 0.96,0.89,0.45 \end{array}\right)$	$\left(\begin{array}{c} 0.25,0.69,0.36\text{,}\\ 0.30,0.89,0.85 \end{array}\right)$	$\left(\begin{array}{c} 0.36,0.85,0.78\text{,}\\ 0.89,0.65,0.39 \end{array}\right)$
$V_{3}$	$\left(\begin{array}{c} 0.96,0.39,0.50\text{,}\\ 0.56,0.39,0.30 \end{array}\right)$	$\left(\begin{array}{c} 0.53,0.23,0.25\text{,}\\ 0.23,0.89,0.23 \end{array}\right)$	$\left(\begin{array}{c} 0.63,0.37,0.25\text{,}\\ 0.96,0.89,0.36 \end{array}\right)$	$\left(\begin{array}{c} 0.78,0.25,0.5\text{,}\\ 0.96,0.89,0.89 \end{array}\right)$
$V_{4}$	$\left(\begin{array}{c} 0.12,0.36,0.50\text{,}\\ 0.85,0.78,0.30 \end{array}\right)$	$\left(\begin{array}{c} 0.78,0.56,0.23\text{,}\\ 0.23,0.96,0.52 \end{array}\right)$	$\left(\begin{array}{c} 0.89,0.65,0.23\text{,}\\ 0.14,0.56,0.47 \end{array}\right)$	$\left(\begin{array}{c} 0.63,0.69,0.57\text{,}\\ 0.85,0.69,0.33 \end{array}\right)$

**Table 3:** Decision matrix $D^{3}$.

**Alternatives**	$J_{1}$	$J_{2}$	$J_{3}$	$J_{4}$
$V_{1}$	$\left(\begin{array}{c} 0.85,0.96,0.08\\ 0.65,0.65,0.19 \end{array}\right)$	$\left(\begin{array}{c} 0.85,0.10,0.56\text{,}\\ 0.48,0.80,0.39 \end{array}\right)$	$\left(\begin{array}{c} 0.54,0.89,0.32\text{,}\\ 0.89,0.56,0.23 \end{array}\right)$	$\left(\begin{array}{c} 0.89,0.30,0.50\text{,}\\ 0.25,0.50,0.30 \end{array}\right)$
$V_{2}$	$\left(\begin{array}{c} 0.78,0.21,0.89\text{,}\\ 0.12,0.98,0.45 \end{array}\right)$	$\left(\begin{array}{c} 0.48,0.29,0.52\text{,}\\ 0.68,0.78,0.35 \end{array}\right)$	$\left(\begin{array}{c} 0.36,0.74,0.56\text{,}\\ 0.23,0.89,0.25 \end{array}\right)$	$\left(\begin{array}{c} 0.65,0.30,0.50\text{,}\\ 0.30,0.50,0.30 \end{array}\right)$
$V_{3}$	$\left(\begin{array}{c} 0.41,0.85,0.78\text{,}\\ 0.01,0.02,0.69 \end{array}\right)$	$\left(\begin{array}{c} 0.28,0.69,0.57\text{,}\\ 0.69,0.85,0.32 \end{array}\right)$	$\left(\begin{array}{c} 0.78,0.63,0.74\text{,}\\ 0.23,0.65,0.25 \end{array}\right)$	$\left(\begin{array}{c} 0.23,0.30,0.50\text{,}\\ 0.39,0.50,0.30 \end{array}\right)$
$V_{4}$	$\left(\begin{array}{c} 0.78,0.30,0.56\text{,}\\ 0.03,0.09,0.11 \end{array}\right)$	$\left(\begin{array}{c} 0.62,0.69,0.58\text{,}\\ 0.96,0.85,0.35 \end{array}\right)$	$\left(\begin{array}{c} 0.32,0.56,0.23\text{,}\\ 0.25,0.58,0.52 \end{array}\right)$	$\left(\begin{array}{c} 0.32,0.30,0.50\text{,}\\ 0.56,0.85,0.85 \end{array}\right)$

**Table 4:** Decision matrix $D^{4}$.

**Alternatives**	$J_{1}$	$J_{2}$	$J_{3}$	$J_{4}$
$V_{1}$	$\left(\begin{array}{c} 0.52,0.89,0.41\text{,}\\ 0.27,0.43,0.96 \end{array}\right)$	$\left(\begin{array}{c} 0.96,0.24,0.28\text{,}\\ 0.30,0.50,0.41 \end{array}\right)$	$\left(\begin{array}{c} 0.25,0.24,0.37\text{,}\\ 0.46,0.26,0.37 \end{array}\right)$	$\left(\begin{array}{c} 0.96,0.96,0.34\text{,}\\ 0.24,0.74,0.96 \end{array}\right)$
$V_{2}$	$\left(\begin{array}{c} 0.50,0.30,0.89\text{,}\\ 0.96,0.58,0.39 \end{array}\right)$	$\left(\begin{array}{c} 0.74,0.74,0.50\text{,}\\ 0.38,0.69,0.32 \end{array}\right)$	$\left(\begin{array}{c} 0.28,0.87,0.21\text{,}\\ 0.98,0.28,0.38 \end{array}\right)$	$\left(\begin{array}{c} 0.96,0.96,0.36\text{,}\\ 0.85,0.74,0.96 \end{array}\right)$
$V_{3}$	$\left(\begin{array}{c} 0.21,0.78,0.27\text{,}\\ 0.79,0.28,0.39 \end{array}\right)$	$\left(\begin{array}{c} 0.21,0.96,0.32\text{,}\\ 0.30,0.28,0.27 \end{array}\right)$	$\left(\begin{array}{c} 0.67,0.15,0.50\text{,}\\ 0.38,0.41,0.97 \end{array}\right)$	$\left(\begin{array}{c} 0.74,0.52,0.96\text{,}\\ 0.96,0.74,0.96 \end{array}\right)$
$V_{4}$	$\left(\begin{array}{c} 0.89,0.38,0.52\text{,}\\ 0.25,0.58,0.96 \end{array}\right)$	$\left(\begin{array}{c} 0.28,0.74,0.25\text{,}\\ 0.87,0.52,0.32 \end{array}\right)$	$\left(\begin{array}{c} 0.25,0.96,0.78\text{,}\\ 0.24,0.36,0.69 \end{array}\right)$	$\left(\begin{array}{c} 0.96,0.21,0.96\text{,}\\ 0.8,0.74,0.25 \end{array}\right)$

"Cost" is a cost-type criterion because it directly relates to the financial expenses associated with implementing the technology solutions offered by the vendors. "Technology expertise", "customer support", and "innovative factors" are benefit-type criteria because they represent the qualitative aspects that contribute to the overall value or benefit derived from the technology solutions: "Technology expertise" evaluates the competency and proficiency of the vendors in applying technology to solve problems, which directly contributes to the value of the solutions. "Customer support" reflects the level of assistance and service provided by the vendors, which enhances the overall value of the vendor's offerings. "Innovative factors" assesses the degree of innovation and potential for future growth and competitiveness that the vendors' solutions offer, which contributes to their overall value. Table 5, Table 6, Table 7, Table 8 provide the normalized decision matrix for evaluating Digital Transformation (Vendor).

**Table 5:** Normalized decision matrix $R^{\left(1\right)}$.

**Alternatives**	$J_{1}$	$J_{2}$	$J_{3}$	$J_{4}$
$V_{1}$	$\left(\begin{array}{c} 0.56,0.63,0.25\text{,}\\ 0.45,0.45,0.96 \end{array}\right)$	$\left(\begin{array}{c} 0.45,0.45,0.89\text{,}\\ 0.56,0.58,0.58 \end{array}\right)$	$\left(\begin{array}{c} 0.25,0.56,0.85\text{,}\\ 0.25,0.56,0.85 \end{array}\right)$	$\left(\begin{array}{c} 0.96,0.85,0.74\text{,}\\ 0.52,0.63,0.63 \end{array}\right)$
$V_{2}$	$\left(\begin{array}{c} 0.36,0.99,0.52\text{,}\\ 0.85,0.25,0.85 \end{array}\right)$	$\left(\begin{array}{c} 0.36,0.56,0.85\text{,}\\ 0.58,0.56,0.36 \end{array}\right)$	$\left(\begin{array}{c} 0.25,0.25,0.25\text{,}\\ 0.89,0.25,0.25 \end{array}\right)$	$\left(\begin{array}{c} 0.25,0.36,0.96\text{,}\\ 0.74,0.14,0.25 \end{array}\right)$
$V_{3}$	$\left(\begin{array}{c} 0.45,0.26,0.23\text{,}\\ 0.25,0.75,0.16 \end{array}\right)$	$\left(\begin{array}{c} 0.89,0.78,0.58\text{,}\\ 0.69,0.78,0.98 \end{array}\right)$	$\left(\begin{array}{c} 0.25,0.38,0.23\text{,}\\ 0.21,0.45,0.78 \end{array}\right)$	$\left(\begin{array}{c} 0.25,0.36,0.36\text{,}\\ 0.36,0.85,0.85 \end{array}\right)$
$V_{4}$	$\left(\begin{array}{c} 0.25,0.96,0.45\text{,}\\ 0.45,0.65,0.85 \end{array}\right)$	$\left(\begin{array}{c} 0.85,0.85,0.78\text{,}\\ 0.65,0.85,0.35 \end{array}\right)$	$\left(\begin{array}{c} 0.56,0.23,0.56\text{,}\\ 0.89,0.23,0.36 \end{array}\right)$	$\left(\begin{array}{c} 0.36,0.96,0.14\text{,}\\ 0.75,0.25,0.75 \end{array}\right)$

**Table 6:** Normalized decision matrix $R^{\left(2\right)}$.

**Alternatives**	$J_{1}$	$J_{2}$	$J_{3}$	$J_{4}$
$V_{1}$	$\left(\begin{array}{c} 0.85,0.39,0.23\text{,}\\ 0.32,0.50,0.63 \end{array}\right)$	$\left(\begin{array}{c} 0.23,0.12,0.54\text{,}\\ 0.23,0.74,0.60 \end{array}\right)$	$\left(\begin{array}{c} 0.85,0.8,0.96\text{,}\\ 0.25,0.36,0.30 \end{array}\right)$	$\left(\begin{array}{c} 0.69,0.36,0.36\text{,}\\ 0.74,0.32,0.69 \end{array}\right)$
$V_{2}$	$\left(\begin{array}{c} 0.85,0.37,0.50\text{,}\\ 0.12,0.78,0.30 \end{array}\right)$	$\left(\begin{array}{c} 0.85,0.96,0.85\text{,}\\ 0.45,0.89,0.96 \end{array}\right)$	$\left(\begin{array}{c} 0.25,0.69,0.36\text{,}\\ 0.30,0.89,0.85 \end{array}\right)$	$\left(\begin{array}{c} 0.36,0.85,0.78\text{,}\\ 0.89,0.65,0.39 \end{array}\right)$
$V_{3}$	$\left(\begin{array}{c} 0.96,0.39,0.50\text{,}\\ 0.56,0.39,0.30 \end{array}\right)$	$\left(\begin{array}{c} 0.25,0.23,0.53\text{,}\\ 0.23,0.89,0.23 \end{array}\right)$	$\left(\begin{array}{c} 0.63,0.37,0.25\text{,}\\ 0.96,0.89,0.36 \end{array}\right)$	$\left(\begin{array}{c} 0.78,0.25,0.5\text{,}\\ 0.96,0.89,0.89 \end{array}\right)$
$V_{4}$	$\left(\begin{array}{c} 0.12,0.36,0.50\text{,}\\ 0.85,0.78,0.30 \end{array}\right)$	$\left(\begin{array}{c} 0.23,0.56,0.78\text{,}\\ 0.52,0.96,0.23 \end{array}\right)$	$\left(\begin{array}{c} 0.89,0.65,0.23\text{,}\\ 0.14,0.56,0.47 \end{array}\right)$	$\left(\begin{array}{c} 0.63,0.69,0.57\text{,}\\ 0.85,0.69,0.33 \end{array}\right)$

**Table 7:** Normalized decision matrix $R^{\left(3\right)}$.

**Alternatives**	$J_{1}$	$J_{2}$	$J_{3}$	$J_{4}$
$V_{1}$	$\left(\begin{array}{c} 0.85,0.96,0.08\\ 0.65,0.65,0.19 \end{array}\right)$	$\left(\begin{array}{c} 0.56,0.10,0.85\text{,}\\ 0.39,0.80,0.48 \end{array}\right)$	$\left(\begin{array}{c} 0.54,0.89,0.32\text{,}\\ 0.89,0.56,0.23 \end{array}\right)$	$\left(\begin{array}{c} 0.89,0.30,0.50\text{,}\\ 0.25,0.50,0.30 \end{array}\right)$
$V_{2}$	$\left(\begin{array}{c} 0.78,0.21,0.89\text{,}\\ 0.12,0.98,0.45 \end{array}\right)$	$\left(\begin{array}{c} 0.52,0.29,0.48\text{,}\\ 0.35,0.78,0.68 \end{array}\right)$	$\left(\begin{array}{c} 0.36,0.74,0.56\text{,}\\ 0.23,0.89,0.25 \end{array}\right)$	$\left(\begin{array}{c} 0.65,0.30,0.50\text{,}\\ 0.30,0.50,0.30 \end{array}\right)$
$V_{3}$	$\left(\begin{array}{c} 0.41,0.85,0.78\text{,}\\ 0.01,0.02,0.69 \end{array}\right)$	$\left(\begin{array}{c} 0.57,0.69,0.28\text{,}\\ 0.32,0.85,0.69 \end{array}\right)$	$\left(\begin{array}{c} 0.78,0.63,0.74\text{,}\\ 0.23,0.65,0.25 \end{array}\right)$	$\left(\begin{array}{c} 0.23,0.30,0.50\text{,}\\ 0.39,0.50,0.30 \end{array}\right)$
$V_{4}$	$\left(\begin{array}{c} 0.78,0.30,0.56\text{,}\\ 0.03,0.09,0.11 \end{array}\right)$	$\left(\begin{array}{c} 0.58,0.69,0.62\text{,}\\ 0.35,0.85,0.96 \end{array}\right)$	$\left(\begin{array}{c} 0.32,0.56,0.23\text{,}\\ 0.25,0.58,0.52 \end{array}\right)$	$\left(\begin{array}{c} 0.32,0.30,0.50\text{,}\\ 0.56,0.85,0.85 \end{array}\right)$

**Table 8:** Normalized decision matrix $R^{\left(4\right)}$.

**Alternatives**	$J_{1}$	$J_{2}$	$J_{3}$	$J_{4}$
$V_{1}$	$\left(\begin{array}{c} 0.52,0.89,0.41\text{,}\\ 0.27,0.43,0.96 \end{array}\right)$	$\left(\begin{array}{c} 0.28,0.24,0.96\text{,}\\ 0.41,0.50,0.30 \end{array}\right)$	$\left(\begin{array}{c} 0.25,0.24,0.37\text{,}\\ 0.46,0.26,0.37 \end{array}\right)$	$\left(\begin{array}{c} 0.96,0.96,0.34\text{,}\\ 0.24,0.74,0.96 \end{array}\right)$
$V_{2}$	$\left(\begin{array}{c} 0.50,0.30,0.89\text{,}\\ 0.96,0.58,0.39 \end{array}\right)$	$\left(\begin{array}{c} 0.50,0.74,0.74\text{,}\\ 0.32,0.69,0.38 \end{array}\right)$	$\left(\begin{array}{c} 0.28,0.87,0.21\text{,}\\ 0.98,0.28,0.38 \end{array}\right)$	$\left(\begin{array}{c} 0.96,0.96,0.36\text{,}\\ 0.85,0.74,0.96 \end{array}\right)$
$V_{3}$	$\left(\begin{array}{c} 0.21,0.78,0.27\text{,}\\ 0.79,0.28,0.39 \end{array}\right)$	$\left(\begin{array}{c} 0.32,0.96,0.21\text{,}\\ 0.27,0.28,0.30 \end{array}\right)$	$\left(\begin{array}{c} 0.67,0.15,0.50\text{,}\\ 0.38,0.41,0.97 \end{array}\right)$	$\left(\begin{array}{c} 0.74,0.52,0.96\text{,}\\ 0.96,0.74,0.96 \end{array}\right)$
$V_{4}$	$\left(\begin{array}{c} 0.89,0.38,0.52\text{,}\\ 0.25,0.58,0.96 \end{array}\right)$	$\left(\begin{array}{c} 0.25,0.74,0.28\text{,}\\ 0.32,0.52,0.87 \end{array}\right)$	$\left(\begin{array}{c} 0.25,0.96,0.78\text{,}\\ 0.24,0.36,0.69 \end{array}\right)$	$\left(\begin{array}{c} 0.96,0.21,0.96\text{,}\\ 0.8,0.74,0.25 \end{array}\right)$

The entropy values for each property are ascertained using Equation (19) and are as follows: $E_{1}=0.8645$, $E_{2}=0.7114$, $E_{3}=0.7663$, and $E_{4}=0.8057$. We may derive the weight vector ω=(0.0719,0.3664,0.3040,0.2577) by using Equation (20). To get the overall values for each alternative, we integrate the values in matrix R using this weight vector ω and the suggested operators. The results obtained are summarised in Table 9.

Calculate the total performance score values for the alternative $V_{i}$ using the given data and the ST−q−SFRWA operator to combine all the individual performance values $r_{j}$ for i between 1 and 4 and j between 1 and 4 as shown below:

$$r_{1}=\left(\begin{array}{c} 0.6534,0.7482,0.2642,\\ 0.8564,0.3257,0.7856, \end{array}\right),r_{2}=\left(\begin{array}{c} 0.5684,0.4586,0.2536,\\ 0.8796,0.5264,0.3667 \end{array}\right),r_{3}=\left(\begin{array}{c} 0.5674,0.9687,0.6378,\\ 0.7856,0.2564,0.5698 \end{array}\right),\text{and}\;r_{4}=\left(\begin{array}{c} 0.8689,0.5874,0.8569,\\ 0.7458,0.6532,0.8745 \end{array}\right)\text{.}$$

By using Equation (21) we get $Sco\left(r_{1}\right)=0.6501$, $Sco\left(r_{2}\right)=0.8521$, $Sco\left(r_{3}\right)=0.4324$, and $Sco\left(r_{4}\right)=0.4305$. Using the score values, we can establish the ranking order of the available alternatives as follows: $V_{2}≻V_{4}≻V_{3}≻V_{1}$. For these assessed alternatives, Table 10 provides a succinct illustration of the scores values and the ensuing ranking order utilizing the ST−q−SFRWA, ST−q−SFRWG, ST−q−SFROWA, and ST−q−SFROWG operators.

**Table 9:** ST−q−SFRWA operator's aggregated expert values (q=3).

**Alternatives**	$J_{1}$	$J_{2}$	$J_{3}$	$J_{4}$
$V_{1}$	$\left(\begin{array}{c} 0.25,0.23,0.85\text{,}\\ 0.52,0.85,0.36 \end{array}\right)$	$\left(\begin{array}{c} 0.76,0.52,0.74\text{,}\\ 0.74,0.52,0.69 \end{array}\right)$	$\left(\begin{array}{c} 0.25,0.85,0.52\text{,}\\ 0.85,0.23,0.25 \end{array}\right)$	$\left(\begin{array}{c} 0.96,0.63,0.63\text{,}\\ 0.78,0.63,0.96 \end{array}\right)$
$V_{2}$	$\left(\begin{array}{c} 0.96,0.25,0.25\text{,}\\ 0.250,0.45,0.63 \end{array}\right)$	$\left(\begin{array}{c} 0.41,0.36,0.96\text{,}\\ 0.76,0.36,0.96 \end{array}\right)$	$\left(\begin{array}{c} 0.14,0.74,0.96\text{,}\\ 0.85,0.12,0.25 \end{array}\right)$	$\left(\begin{array}{c} 0.85,0.74,0.98\text{,}\\ 0.93,0.41,0.63 \end{array}\right)$
$V_{3}$	$\left(\begin{array}{c} 0.25,0.85,0.25\text{,}\\ 0.85,0.85,0.45 \end{array}\right)$	$\left(\begin{array}{c} 0.75,0.74,0.52\text{,}\\ 0.41,0.63,0.96 \end{array}\right)$	$\left(\begin{array}{c} 0.12,0.63,0.36\text{,}\\ 0.25,0.45,0.78 \end{array}\right)$	$\left(\begin{array}{c} 0.32,0.74,0.96\text{,}\\ 0.74,0.85,0.63 \end{array}\right)$
$V_{4}$	$\left(\begin{array}{c} 0.26,0.96,0.25\text{,}\\ 0.25,0.96,0.85 \end{array}\right)$	$\left(\begin{array}{c} 0.47,0.96,0.96\text{,}\\ 0.85,0.25,0.63 \end{array}\right)$	$\left(\begin{array}{c} 0.25,0.63,0.87\text{,}\\ 0.74,0.65,0.36 \end{array}\right)$	$\left(\begin{array}{c} 0.25,0.74,0.74\text{,}\\ 0.96,0.23,0.98 \end{array}\right)$

**Table 10:** Alternatives scores and sequence of ranking.

Operators	Score values				Ranking
	$V_{1}$	$V_{2}$	$V_{3}$	$V_{4}$	
ST−q−SFRWA	0.6501	0.8521	0.4324	0.4305	$V_{2}≻V_{1}≻V_{3}≻V_{4}$
ST−q−SFRGA	0.6524	0.8648	0.5874	0.4236	$V_{2}≻V_{1}≻V_{3}≻V_{4}$
ST−q−SFROWA	0.5924	0.8902	0.4136	0.3674	$V_{2}≻V_{1}≻V_{3}≻V_{4}$
ST−q−SFR0WG	0.6472	0.9376	0.3897	0.2745	$V_{2}≻V_{1}≻V_{3}≻V_{4}$

This study also explores the applicability of these operators in scenarios where decision-makers want to tailor their choice aggregation methods according to their personal preferences. Table 10 showcases the outcomes when different operators are used, illustrating how decision-makers can enhance their decision-making by considering both assigned values and expert opinions simultaneously. It is clear from the preceding discussion that the recommended aggregation operators provide decision-makers with a more adaptable framework for identifying suitable alternatives. Furthermore, compared to existing aggregation approaches, these operators provide greater generality. This suggests that a greater range of decision-making scenarios can be handled by the recommended operators, who also offer greater flexibility and relevance in several contexts. By offering a more adaptable and inclusive framework, these aggregation operators empower decision-makers to make well-informed choices that are consistent with their requirements and preferences. Moreover, the generalizability of these operators ensures their effectiveness in a wide range of decision domains, enhancing the overall robustness and dependability of the decision-making process.

**Fig. 3:**
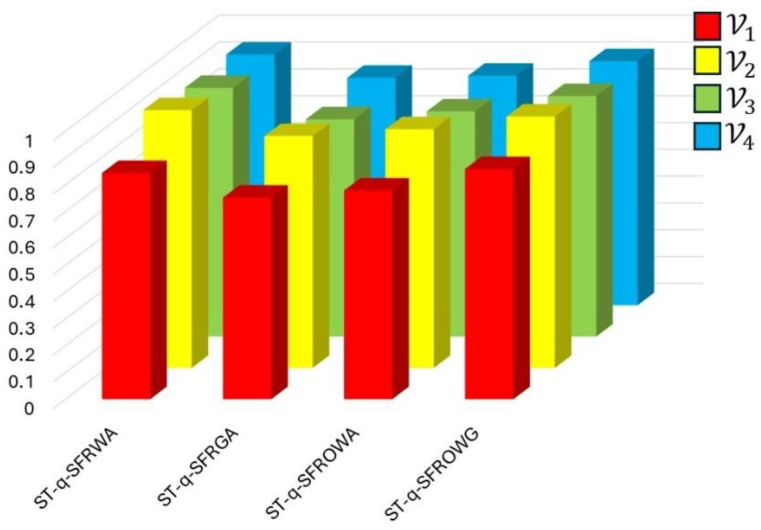
A graphical representation of score values with multiple alternatives.

The statistics included in Table 10 are shown visually in Fig. 3.

**Example 2.** A company is looking to choose a cloud service provider for their digital transformation initiative. They have identified four key criteria for evaluation: “Infrastructure Scalability,” “Security,” “Service Reliability,” and “Cost Efficiency”. A panel of four experts will assign weights to these criteria and will use a decision matrix and decision tree to make the selection.

**Scenario: Selecting a Cloud Service Provider for Digital Transformation**.

**Candidates (Cloud Service Providers):**

$$V_{1}=\text{Amazon}\;\text{Web}\;\text{Service.}$$

$$V_{2}=\text{Microsoft}\;\text{Azure.}$$

$$V_{3}=\text{Google}\;\text{Cloud}\;\text{Platform.}$$

$$V_{4}=\text{IBM}\;\text{Cloud}$$

**Criteria (Attributes):**

$$J_{1}=\text{Infrastructure}\;\text{Scalability}$$

$$J_{2}=\text{Security}$$

$$J_{3}=\text{Service}\;\text{Reliability}$$

$$J_{4}=\text{Cost}\;\text{Efficiency}$$

**Weight Vector (Expert Opinion):**

w=(0.3,0.1,0.2,0.4).

The weight vector presents the importance of each criterion as determined by a group of experts. Now let's create a decision matrix $D^{\left(k\right)}=\left(\alpha_{ij}^{\left(k\right)}\right)$ for each vendor (k=1,2,3,4), The developed aggregation operators—in particular, ST-q-SFRWA—will be employed. The decision experts assessed each assessment report AI using q-SFRS considering the pertinent criteria. The assessments offered by the three experts, $D^{1}$, $D^{2}$, and $D^{3}$, are shown in Table 11, Table 12, Table 13, Table 14, respectively.

**Table 11:** Expert $D^{1}$'s judgment details.

Alternatives	$J_{1}$	$J_{2}$	$J_{3}$	$J_{4}$	$J_{5}$
$V_{1}$	$\left(\begin{array}{c} 0.52,0.29,0.91\text{,}\\ 0.96,0.38,0.98 \end{array}\right)$	$\left(\begin{array}{c} 0.56,0.25,0.15\text{,}\\ 0.56,0.96,0.26 \end{array}\right)$	$\left(\begin{array}{c} 0.62,0.36,0.12\text{,}\\ 0.64,0.48,0.23 \end{array}\right)$	$\left(\begin{array}{c} 0.69,0.85,0.96\text{,}\\ 0.74,0.83,0.38 \end{array}\right)$	$\left(\begin{array}{c} 0.41,0.74,0.96\text{,}\\ 0.44,0.63,0.85 \end{array}\right)$
$V_{2}$	$\left(\begin{array}{c} 0.12,0.47,0.93\text{,}\\ 0.36,0.56,0.95 \end{array}\right)$	$\left(\begin{array}{c} 0.89,0.36,0.65\text{,}\\ 0.53,0.74,0.25 \end{array}\right)$	$\left(\begin{array}{c} 0.65,0.82,0.45\text{,}\\ 0.69,0.90,0.67 \end{array}\right)$	$\left(\begin{array}{c} 0.56,0.82,0.52\text{,}\\ 0.25,0.71,0.25 \end{array}\right)$	$\left(\begin{array}{c} 0.49,0.36,0.74\text{,}\\ 0.43,0.85,0.65 \end{array}\right)$
$V_{3}$	$\left(\begin{array}{c} 0.14,0.65,0.82\text{,}\\ 0.74,0.74,0.84 \end{array}\right)$	$\left(\begin{array}{c} 0.52,0.96,0.74\text{,}\\ 0.57,0.96,0.63 \end{array}\right)$	$\left(\begin{array}{c} 0.67,0.78,0.89\text{,}\\ 0.62,0.56,0.98 \end{array}\right)$	$\left(\begin{array}{c} 0.26,0.98,0.25\text{,}\\ 0.36,0.93,0.25 \end{array}\right)$	$\left(\begin{array}{c} 0.42,0.74,0.53\text{,}\\ 0.43,0.36,0.48 \end{array}\right)$
$V_{4}$	$\left(\begin{array}{c} 0.85,0.83,0.81\text{,}\\ 0.52,0.92,0.89 \end{array}\right)$	$\left(\begin{array}{c} 0.53,0.74,0.24\text{,}\\ 0.58,0.25,0.74 \end{array}\right)$	$\left(\begin{array}{c} 0.63,0.34,0.25\text{,}\\ 0.65,0.12,0.25 \end{array}\right)$	$\left(\begin{array}{c} 0.45,0.93,0.36\text{,}\\ 0.98,0.74,0.74 \end{array}\right)$	$\left(\begin{array}{c} 0.49,0.85,0.32\text{,}\\ 0.25,0.96,0.32 \end{array}\right)$

**Table 12:** Expert $D^{2}$'s judgment details.

**Alternatives**	$J_{1}$	$J_{2}$	$J_{3}$	$J_{4}$	$J_{5}$
$V_{1}$	$\left(\begin{array}{c} 0.85,0.18,0.71\text{,}\\ 0.85,0.29,0.79 \end{array}\right)$	$\left(\begin{array}{c} 0.56,0.96,0.96\text{,}\\ 0.25,0.56,0.36 \end{array}\right)$	$\left(\begin{array}{c} 0.12,0.47,0.93\text{,}\\ 0.36,0.56,0.95 \end{array}\right)$	$\left(\begin{array}{c} 0.96,0.82,0.75\text{,}\\ 0.36,0.91,0.75 \end{array}\right)$	$\left(\begin{array}{c} 0.52,0.29,0.91\text{,}\\ 0.96,0.38,0.98 \end{array}\right)$
$V_{2}$	$\left(\begin{array}{c} 0.96,0.40,0.72\text{,}\\ 0.63,0.51,0.78 \end{array}\right)$	$\left(\begin{array}{c} 0.67,0.78,0.89\text{,}\\ 0.62,0.56,0.98 \end{array}\right)$	$\left(\begin{array}{c} 0.96,0.40,0.72\text{,}\\ 0.63,0.51,0.78 \end{array}\right)$	$\left(\begin{array}{c} 0.50,0.30,0.50\text{,}\\ 0.30,0.50,0.30 \end{array}\right)$	$\left(\begin{array}{c} 0.13,0.35,0.25\text{,}\\ 0.18,0.25,0.85 \end{array}\right)$
$V_{3}$	$\left(\begin{array}{c} 0.25,0.62,0.76\text{,}\\ 0.94,0.73,0.75 \end{array}\right)$	$\left(\begin{array}{c} 0.55,0.30,0.45\text{,}\\ 0.36,0.50,0.98 \end{array}\right)$	$\left(\begin{array}{c} 0.50,0.30,0.70\text{,}\\ 0.30,0.50,0.30 \end{array}\right)$	$\left(\begin{array}{c} 0.96,0.40,0.72\text{,}\\ 0.63,0.51,0.78 \end{array}\right)$	$\left(\begin{array}{c} 0.26,0.98,0.25\text{,}\\ 0.36,0.93,0.25 \end{array}\right)$
$V_{4}$	$\left(\begin{array}{c} 0.96,0.82,0.75\text{,}\\ 0.36,0.91,0.75 \end{array}\right)$	$\left(\begin{array}{c} 0.53,0.37,0.56\text{,}\\ 0.37,0.53,0.30 \end{array}\right)$	$\left(\begin{array}{c} 0.13,0.35,0.25\text{,}\\ 0.18,0.25,0.85 \end{array}\right)$	$\left(\begin{array}{c} 0.50,0.30,0.50\text{,}\\ 0.30,0.50,0.30 \end{array}\right)$	$\left(\begin{array}{c} 0.41,0.74,0.96\text{,}\\ 0.44,0.63,0.85 \end{array}\right)$

**Table 13:** Expert $D^{3}$'s judgment details.

**Alternatives**	$J_{1}$	$J_{2}$	$J_{3}$	$J_{4}$	$J_{5}$
$V_{1}$	$\left(\begin{array}{c} 0.96,0.96,0.68\text{,}\\ 0.25,0.89,0.56 \end{array}\right)$	$\left(\begin{array}{c} 0.50,0.30,0.50\text{,}\\ 0.30,0.50,0.30 \end{array}\right)$	$\left(\begin{array}{c} 0.50,0.90,0.30\text{,}\\ 0.30,0.90,0.70 \end{array}\right)$	$\left(\begin{array}{c} 0.96,0.40,0.72\text{,}\\ 0.63,0.51,0.78 \end{array}\right)$	$\left(\begin{array}{c} 0.59,0.32,0.53\text{,}\\ 0.32,0.90,0.35 \end{array}\right)$
$V_{2}$	$\left(\begin{array}{c} 0.36,0.72,0.78\text{,}\\ 0.96,0.69,0.25 \end{array}\right)$	$\left(\begin{array}{c} 0.26,0.98,0.25\text{,}\\ 0.36,0.93,0.25 \end{array}\right)$	$\left(\begin{array}{c} 0.41,0.74,0.96\text{,}\\ 0.44,0.63,0.85 \end{array}\right)$	$\left(\begin{array}{c} 0.20,0.30,0.20\text{,}\\ 0.30,0.20,0.30 \end{array}\right)$	$\left(\begin{array}{c} 0.56,0.30,0.50\text{,}\\ 0.90,0.20,0.60 \end{array}\right)$
$V_{3}$	$\left(\begin{array}{c} 0.78,0.59,0.62\text{,}\\ 0.25,0.49,0.25 \end{array}\right)$	$\left(\begin{array}{c} 0.67,0.78,0.89\text{,}\\ 0.62,0.56,0.98 \end{array}\right)$	$\left(\begin{array}{c} 0.96,0.40,0.72\text{,}\\ 0.63,0.51,0.78 \end{array}\right)$	$\left(\begin{array}{c} 0.96,0.82,0.75\text{,}\\ 0.36,0.91,0.75 \end{array}\right)$	$\left(\begin{array}{c} 0.52,0.30,0.59\text{,}\\ 0.90,0.50,0.30 \end{array}\right)$
$V_{4}$	$\left(\begin{array}{c} 0.13,0.35,0.25\text{,}\\ 0.18,0.25,0.85 \end{array}\right)$	$\left(\begin{array}{c} 0.52,0.29,0.91\text{,}\\ 0.96,0.38,0.98 \end{array}\right)$	$\left(\begin{array}{c} 0.50,0.30,0.50\text{,}\\ 0.30,0.50,0.30 \end{array}\right)$	$\left(\begin{array}{c} 0.12,0.47,0.93\text{,}\\ 0.36,0.56,0.95 \end{array}\right)$	$\left(\begin{array}{c} 0.41,0.74,0.96\text{,}\\ 0.44,0.63,0.85 \end{array}\right)$

"Cost Efficiency" is a cost-type criterion because it directly relates to the financial expenses associated with using the cloud service provider. It focuses on how economically feasible the provider's services are for the company. "Infrastructure Scalability," "Security," and "Service Reliability" are benefit-type criteria because they represent qualitative aspects that contribute to the overall value or benefit derived from the cloud service provider: "Infrastructure Scalability" evaluates the provider's ability to scale resources according to the company's needs, which directly contributes to the flexibility and adaptability of the cloud services. "Security" assesses the level of security measures implemented by the provider to protect the company's data and infrastructure, enhancing the overall value and trustworthiness of the services. "Service Reliability" reflects the provider's track record in delivering consistent and dependable services, which enhances the overall reliability and usability of the cloud services. Table 14, Table 15, Table 16 provide the normalized decision matrix for evaluating Cloud Service Providers for Digital Transformation.

**Table 14:** Normalized decision matrix $R^{\left(1\right)}$.

Alternatives	$J_{1}$	$J_{2}$	$J_{3}$	$J_{4}$	$J_{5}$
$V_{1}$	$\left(\begin{array}{c} 0.91,0.29,0.52\text{,}\\ 0.98,0.38,0.96 \end{array}\right)$	$\left(\begin{array}{c} 0.56,0.25,0.15\text{,}\\ 0.56,0.96,0.26 \end{array}\right)$	$\left(\begin{array}{c} 0.62,0.36,0.12\text{,}\\ 0.64,0.48,0.23 \end{array}\right)$	$\left(\begin{array}{c} 0.69,0.85,0.96\text{,}\\ 0.74,0.83,0.38 \end{array}\right)$	$\left(\begin{array}{c} 0.41,0.74,0.96\text{,}\\ 0.44,0.63,0.85 \end{array}\right)$
$V_{2}$	$\left(\begin{array}{c} 0.93,0.47,0.12\text{,}\\ 0.95,0.56,0.365 \end{array}\right)$	$\left(\begin{array}{c} 0.89,0.36,0.65\text{,}\\ 0.53,0.74,0.25 \end{array}\right)$	$\left(\begin{array}{c} 0.65,0.82,0.45\text{,}\\ 0.69,0.90,0.67 \end{array}\right)$	$\left(\begin{array}{c} 0.56,0.82,0.52\text{,}\\ 0.25,0.71,0.25 \end{array}\right)$	$\left(\begin{array}{c} 0.49,0.36,0.74\text{,}\\ 0.43,0.85,0.65 \end{array}\right)$
$V_{3}$	$\left(\begin{array}{c} 0.82,0.65,0.14\text{,}\\ 0.84,0.74,0.74 \end{array}\right)$	$\left(\begin{array}{c} 0.52,0.96,0.74\text{,}\\ 0.57,0.96,0.63 \end{array}\right)$	$\left(\begin{array}{c} 0.67,0.78,0.89\text{,}\\ 0.62,0.56,0.98 \end{array}\right)$	$\left(\begin{array}{c} 0.26,0.98,0.25\text{,}\\ 0.36,0.93,0.25 \end{array}\right)$	$\left(\begin{array}{c} 0.42,0.74,0.53\text{,}\\ 0.43,0.36,0.48 \end{array}\right)$
$V_{4}$	$\left(\begin{array}{c} 0.81,0.83,0.85\text{,}\\ 0.89,0.92,0.52 \end{array}\right)$	$\left(\begin{array}{c} 0.53,0.74,0.24\text{,}\\ 0.58,0.25,0.74 \end{array}\right)$	$\left(\begin{array}{c} 0.63,0.34,0.25\text{,}\\ 0.65,0.12,0.25 \end{array}\right)$	$\left(\begin{array}{c} 0.45,0.93,0.36\text{,}\\ 0.98,0.74,0.74 \end{array}\right)$	$\left(\begin{array}{c} 0.49,0.85,0.32\text{,}\\ 0.25,0.96,0.32 \end{array}\right)$

**Table 15:** Normalized decision matrix $R^{\left(2\right)}$.

**Alternatives**	$J_{1}$	$J_{2}$	$J_{3}$	$J_{4}$	$J_{5}$
$V_{1}$	$\left(\begin{array}{c} 0.71,0.18,0.85\text{,}\\ 0.79,0.29,0.85 \end{array}\right)$	$\left(\begin{array}{c} 0.56,0.96,0.96\text{,}\\ 0.25,0.56,0.36 \end{array}\right)$	$\left(\begin{array}{c} 0.12,0.47,0.93\text{,}\\ 0.36,0.56,0.95 \end{array}\right)$	$\left(\begin{array}{c} 0.96,0.82,0.75\text{,}\\ 0.36,0.91,0.75 \end{array}\right)$	$\left(\begin{array}{c} 0.52,0.29,0.91\text{,}\\ 0.96,0.38,0.98 \end{array}\right)$
$V_{2}$	$\left(\begin{array}{c} 0.72,0.40,0.96\text{,}\\ 0.78,0.51,0.63 \end{array}\right)$	$\left(\begin{array}{c} 0.67,0.78,0.89\text{,}\\ 0.62,0.56,0.98 \end{array}\right)$	$\left(\begin{array}{c} 0.96,0.40,0.72\text{,}\\ 0.63,0.51,0.78 \end{array}\right)$	$\left(\begin{array}{c} 0.50,0.30,0.50\text{,}\\ 0.30,0.50,0.30 \end{array}\right)$	$\left(\begin{array}{c} 0.13,0.35,0.25\text{,}\\ 0.18,0.25,0.85 \end{array}\right)$
$V_{3}$	$\left(\begin{array}{c} 0.76,0.62,0.25\text{,}\\ 0.75,0.73,0.94 \end{array}\right)$	$\left(\begin{array}{c} 0.55,0.30,0.45\text{,}\\ 0.36,0.50,0.98 \end{array}\right)$	$\left(\begin{array}{c} 0.50,0.30,0.70\text{,}\\ 0.30,0.50,0.30 \end{array}\right)$	$\left(\begin{array}{c} 0.96,0.40,0.72\text{,}\\ 0.63,0.51,0.78 \end{array}\right)$	$\left(\begin{array}{c} 0.26,0.98,0.25\text{,}\\ 0.36,0.93,0.25 \end{array}\right)$
$V_{4}$	$\left(\begin{array}{c} 0.75,0.82,0.96\text{,}\\ 0.75,0.91,0.36 \end{array}\right)$	$\left(\begin{array}{c} 0.53,0.37,0.56\text{,}\\ 0.37,0.53,0.30 \end{array}\right)$	$\left(\begin{array}{c} 0.13,0.35,0.25\text{,}\\ 0.18,0.25,0.85 \end{array}\right)$	$\left(\begin{array}{c} 0.50,0.30,0.50\text{,}\\ 0.30,0.50,0.30 \end{array}\right)$	$\left(\begin{array}{c} 0.41,0.74,0.96\text{,}\\ 0.44,0.63,0.85 \end{array}\right)$

**Table 16:** Normalized decision matrix $R^{\left(3\right)}$.

**Alternatives**	$J_{1}$	$J_{2}$	$J_{3}$	$J_{4}$	$J_{5}$
$V_{1}$	$\left(\begin{array}{c} 0.68,0.96,0.96\text{,}\\ 0.56,0.89,0.25 \end{array}\right)$	$\left(\begin{array}{c} 0.50,0.30,0.50\text{,}\\ 0.30,0.50,0.30 \end{array}\right)$	$\left(\begin{array}{c} 0.50,0.90,0.30\text{,}\\ 0.30,0.90,0.70 \end{array}\right)$	$\left(\begin{array}{c} 0.96,0.40,0.72\text{,}\\ 0.63,0.51,0.78 \end{array}\right)$	$\left(\begin{array}{c} 0.59,0.32,0.53\text{,}\\ 0.32,0.90,0.35 \end{array}\right)$
$V_{2}$	$\left(\begin{array}{c} 0.78,0.72,0.36\text{,}\\ 0.25,0.69,0.965 \end{array}\right)$	$\left(\begin{array}{c} 0.26,0.98,0.25\text{,}\\ 0.36,0.93,0.25 \end{array}\right)$	$\left(\begin{array}{c} 0.41,0.74,0.96\text{,}\\ 0.44,0.63,0.85 \end{array}\right)$	$\left(\begin{array}{c} 0.20,0.30,0.20\text{,}\\ 0.30,0.20,0.30 \end{array}\right)$	$\left(\begin{array}{c} 0.56,0.30,0.50\text{,}\\ 0.90,0.20,0.60 \end{array}\right)$
$V_{3}$	$\left(\begin{array}{c} 0.62,0.59,0.78\text{,}\\ 0.25,0.49,0.25 \end{array}\right)$	$\left(\begin{array}{c} 0.67,0.78,0.89\text{,}\\ 0.62,0.56,0.98 \end{array}\right)$	$\left(\begin{array}{c} 0.96,0.40,0.72\text{,}\\ 0.63,0.51,0.78 \end{array}\right)$	$\left(\begin{array}{c} 0.96,0.82,0.75\text{,}\\ 0.36,0.91,0.75 \end{array}\right)$	$\left(\begin{array}{c} 0.52,0.30,0.59\text{,}\\ 0.90,0.50,0.30 \end{array}\right)$
$V_{4}$	$\left(\begin{array}{c} 0.25,0.35,0.13\text{,}\\ 0.85,0.25,0.18 \end{array}\right)$	$\left(\begin{array}{c} 0.52,0.29,0.91\text{,}\\ 0.96,0.38,0.98 \end{array}\right)$	$\left(\begin{array}{c} 0.50,0.30,0.50\text{,}\\ 0.30,0.50,0.30 \end{array}\right)$	$\left(\begin{array}{c} 0.12,0.47,0.93\text{,}\\ 0.36,0.56,0.95 \end{array}\right)$	$\left(\begin{array}{c} 0.41,0.74,0.96\text{,}\\ 0.44,0.63,0.85 \end{array}\right)$

According to the information provided, q-spherical fuzzy rough sets are suitable for effectively handling this kind of data. The square sums of 0.7,0.8, 0.9 0.9,0.8, and 0.7 surpass 1 when looking at the pair (0.7,0.8,0.9, 0.9,0.8,0.7). The decision-maker may provide information and effectively manage the q-spherical fuzzy environment when q equals 3 (q = 3). In this setting, $0.7^{3}+0.8^{3}+0.9^{3}≼1\;\text{and}\;0.9^{3}+0.8^{3}+0.7^{3}≼1$ is necessary. Decision-makers must thus utilize the same q value for membership, neutral, and non-membership aspects when making judgments. To overcome these limitations, the proposed aggregation techniques are used in the q-SFR context. Decision-makers in this scenario have access to two factors, q, where q ≽ 1. The Yue [20] model determines the weights for each criterion, which are given as (0.2101,0.1137,0.2439,0.1780,0.1543). Table 17 summarizes the score values and ranking order of the choices for q = 3.

From the previous discussion, it is evident that the existing aggregation operators might be viewed as special instances within the suggested framework. This conclusion suggests that the suggested method offers a more comprehensive and wide-ranging approach compared to the current aggregation operators. From the previous discussion, it is evident that the existing aggregation operators might be viewed as special instances within the suggested framework. This conclusion suggests that the suggested method offers a more comprehensive and wide-ranging approach compared to the current aggregation operators. Furthermore, the information shown in Table 17 demonstrated that, even with the recommended operators, the alternatives' ranking order does not shift. This suggests that any of these operators can be applied at any point throughout the aggregate process without having a substantial impact on the final ranking of the options. Therefore, additional considerations like processing speed or requirements in the decision-making setting could have an impact on the operator's choice.

**Table 17:** The score values and ranking order of alternatives.

Operators	Score values				Ranking orders
	$V_{1}$	$V_{2}$	$V_{3}$	$V_{4}$	
ST−q−SFRWA	0.9948	0.8574	0.8265	0.8321	$V_{1}≻V_{2}≻V_{3}≻V_{4}$
ST−q−SFRWG	0.9634	0.8618	0.8077	0.8182	$V_{1}≻V_{2}≻V_{3}≻V_{4}$
ST−,q−SFROWA	0.8574	0.7952	0.7616	0.7769	$V_{1}≻V_{2}≻V_{3}≻V_{4}$
ST−q−SFROWG	0.9563	0.8563	0.7932	0.8196	$V_{1}≻V_{2}≻V_{3}≻V_{4}$

### 5.2 Effect of q ranking order and score values

$\left(\begin{array}{l} \left({0\leq \left(\mu_{\;\underline{Q}}\left(b\right)\right)}^{q}+{\left(\zeta_{\underline{Q}}\left(b\right)\right)}^{q}+{\left(\upsilon_{\;\underline{Q}}\left(b\right)\right)}^{q}\leq 1\right)\text{,}\\ \left({0\leq \left(\mu_{\;\bar{Q}}\left(b\right)\right)}^{q}+{\left(\zeta_{\;\bar{Q}}\left(b\right)\right)}^{q}+{\left(\upsilon_{\;\bar{Q}}\left(b\right)\right)}^{q}\leq 1\right) \end{array}\right)$, and then by examining the attribute values, a decision-maker can determine which integer parameter, q, is the smallest. For example, while evaluating an alternative, if the attribute values are (0.8,0.7,0.9,0.9,0.8,0.7), one should choose q as 3 or q as 4, as both configurations meet the criterion. However, we employed several values of q in Steps 2 and 5 of the novel approach to solve the case to fully evaluate the effect of parameter q on the experimental results. Table 18 presents the results of these modifications and indicates that $V_{2}$ is at the top, followed by $V_{4}$, $V_{3}$, and finally, $V_{1}$. Notable is the relevance of the best alternative and the unchanging ranking. Table 18 illustrates this point. Specifically, when q equals 1. The alternatives and ratings offered do not adhere to the requirements of either 1 (i.e., under PFRS environment $\left(\begin{array}{l} \left(0\leq \mu_{\;\underline{Q}}\left(b\right)+\zeta_{\underline{Q}}\left(b\right)+\upsilon_{\;\underline{Q}}\left(b\right)\leq 1\right)\text{,}\\ \left(0\leq \mu_{\;\bar{Q}}\left(b\right)+\zeta_{\;\bar{Q}}\left(b\right)+\upsilon_{\;\bar{Q}}\left(b\right)\leq 1\right) \end{array}\right)$) or 2 (i.e., under SFRS environment $\left(\begin{array}{l} \left({0\leq \left(\mu_{\;\underline{Q}}\left(b\right)\right)}^{2}+{\left(\zeta_{\underline{Q}}\left(b\right)\right)}^{2}+{\left(\upsilon_{\;\underline{Q}}\left(b\right)\right)}^{2}\leq 1\right)\text{,}\\ \left({0\leq \left(\mu_{\;\bar{Q}}\left(b\right)\right)}^{2}+{\left(\zeta_{\;\bar{Q}}\left(b\right)\right)}^{2}+{\left(\upsilon_{\;\bar{Q}}\left(b\right)\right)}^{2}\leq 1\right) \end{array}\right)$).

Table 18 shows how, for a range of q-parameter values, the ranking order of the alternatives stays consistent. This consistent ranking provides decision-makers with a robust framework to evaluate test alternatives within a given collection of finite alternatives. This gives decision-makers a secure and adaptable environment, facilitating careful examination and well-informed choices based on the specified parameters.

**Table 18:** Sorting alternatives according to their respective parameter q values.

**Parameter**q	Ranking order	Best alternative
q=1	Unable to determine	__
q=2	Unable to determine	__
q=3	$V_{2}≻V_{1}≻V_{3}≻V_{4}$	$V_{2}$
q=4	$V_{2}≻V_{1}≻V_{3}≻V_{4}$	$V_{2}$
q=5	$V_{2}≻V_{1}≻V_{3}≻V_{4}$	$V_{2}$
q=6	$V_{2}≻V_{1}≻V_{3}≻V_{4}$	$V_{2}$
q=7	$V_{2}≻V_{1}≻V_{3}≻V_{4}$	$V_{2}$
q=8	$V_{2}≻V_{1}≻V_{3}≻V_{4}$	$V_{2}$
q=9	$V_{2}≻V_{1}≻V_{3}≻V_{4}$	$V_{2}$
q=10	$V_{2}≻V_{1}≻V_{3}≻V_{4}$	$V_{2}$
q=11	$V_{2}≻V_{1}≻V_{3}≻V_{4}$	$V_{2}$

### 5.3 Test of validity

To illustrate the adaptability of the proposed technique in various settings, we utilize the evaluation protocols developed by Wang and Trianaphyllou [21] in the following ways.

**Step 1.** Replacing the rating values of less-than-ideal alternatives with those of inferior quality shouldn't affect the identification of the best alternative, preserving the selection that is rated highest, and assuming stable relative weights for the criterion.
**Step 2.** Transitivity should be followed in the procedure.
**Step 3.** When using the same decision-making process for a given problem that has been broken into smaller ones, the initial ranking of the alternatives should be preserved.

**Test of validity utilizing criteria 1.**

The alternatives ranked by using our suggested method are $V_{2}{≻V}_{1}≻V_{3}≻V_{4}$. Based on test criteria 1, we replaced the non-optimal alternative $V_{4}$ with the lowest alternative $V_{4}^{*}$ to evaluate the stability of the suggested method. (0.25,0.93,0.82,0.72,0.42,0.96), (0.25,0.86,0.36,0.32,0.96,0.25), and (0.93,0.83,0.62,0.75,0.36,0.46) were used as the rating values of $L_{4}^{*}\text{.}$ The aggregated score values for the alternatives were as follows after we used our suggested methodology: $Sco\left(L_{2}\right)=0.9613$, $Sco\left(L_{2}\right)=0.8390$, $Sco\left(L_{3}\right)=0.7245$, and $Sco\left(L_{4}^{*}\right)=0.7165$. As a result, $V_{2}≻V_{1}≻V_{3}≻V_{4}^{*}$ is the new ranking order, and the best alternative still adheres to the first suggested strategy. Consequently, our method meets test requirement 1 by producing a consistent result.

**Test of validity employing criteria 1 and 2.**

The fragmented decision-making subcases are regarded as $\left\{V_{1},V_{2},V_{3}\right\}$, $\left\{V_{2},V_{3},V_{4}\right\}$ and $\left\{V_{1},V_{3},V_{4}\right\}$ to assess the validity based on criteria 2 and 3. They rank in the following sequence via the procedures mentioned: $V_{2}≻V_{1}≻V_{3}$, $V_{2}≻V_{3}≻V_{4}$ and $V_{1}≻V_{3}≻V_{4}$. After combining all the findings, the overall ranking appears as $V_{2}≻V_{1}≻V_{3}≻V_{4}$, which is exactly in line with the outcomes of the initial decision-making process. As a result, our suggested strategy meets requirements 2 and 3.

### 5.4 Comparative studies

To show that our suggested mean operator performs better than existing techniques like the Dombi operator [22,23], the averaging operator [8,24], and the geometric operator [25,26], we conducted an inquiry spanning a range of parameter q. This method allows us to determine the best score values and rankings for the different alternatives, as indicated in Table 19, which gives crucial context for comprehending how they compare. Our results show that our proposed strategy is better than any other approach, both in terms of stability and conformance to the state-of-the-art. Additionally, the study highlights the need to use a different computational technique than existing methods, particularly in a diverse situation. This approach leads to more realistic decision-making outcomes and a more logical decision-making process that more closely mimics real-world events by taking into account the consistent precedence between two arguments.

**Table 19:** shows the alternatives' ranking order and score values for a few accepted techniques.

Approaches	Score values				Ranking order
	$V_{1}$	$V_{2}$	$V_{3}$	$V_{4}$	
**Senapati and Yager** [8]	0.4453	0.6578	0.5670	0.6011	$V_{2}≻V_{4}≻V_{3}≻V_{1}$
**Silambarasan** [20]	0.2712	0.4887	0.3169	0.4392	$V_{2}≻V_{4}≻V_{3}≻V_{1}$
**Aydemir and Gunduz** [23]	0.2230	0.3421	0.2518	0.3071	$V_{2}≻V_{4}≻V_{3}≻V_{1}$
**Shit and Ghora** [22]	0.1609	0.3248	0.2319	0.2525	$V_{2}≻V_{4}≻V_{3}≻V_{1}$
**Akram et al.** [24]	0.3288	0.5147	0.3538	0.4266	$V_{2}≻V_{4}≻V_{3}≻V_{1}$
**Chinram** [26]	0.4811	0.7237	0.5381	0.5594	$V_{2}≻V_{4}≻V_{3}≻V_{1}$
**[This Paper]**	0.6501	0.8521	0.4324	0.4205	$V_{2}≻V_{1}≻V_{3}≻V_{4}$

**Fig. 4:**
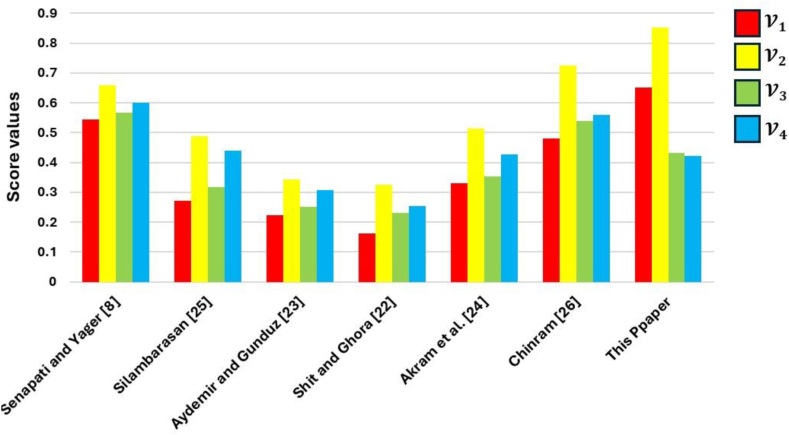
Ranking of alternatives using existing methods.

Fig. 4 showcases the comparative ranking of alternatives, in conjunction with various established approaches. This visual representation offers a clear and concise comparative view of different options, aiding in the assessment of their respective strengths and weaknesses.

The ranking of alternatives across several procedures that provide identical results is displayed in Table 19. However, it's critical to recognize that each strategy has its limitations. For instance, the FFRS techniques restrict decision-makers' ability to consider options within the limitations of $\left(\left({0\leq \left(\mu_{\;\underline{Q}}\left(b\right)\right)}^{3}+{\left(\zeta_{\underline{Q}}\left(b\right)\right)}^{3}+{\left(\upsilon_{\;\underline{Q}}\left(b\right)\right)}^{3}\leq 1\right),\left({0\leq \left(\mu_{\;\bar{Q}}\left(b\right)\right)}^{3}+{\left(\zeta_{\;\bar{Q}}\left(b\right)\right)}^{3}+{\left(\upsilon_{\;\bar{Q}}\left(b\right)\right)}^{3}\leq 1\right)\right)\text{.}$ On the other hand, decision-makers are directed to assess alternatives in the q-rung orthopair fuzzy technique provided that $\left(\left({0\leq \left(\mu_{\;\underline{Q}}\left(b\right)\right)}^{q}+{\left(\zeta_{\underline{Q}}\left(b\right)\right)}^{q}+{\left(\upsilon_{\;\underline{Q}}\left(b\right)\right)}^{q}\leq 1\right),\left({0\leq \left(\mu_{\;\bar{Q}}\left(b\right)\right)}^{q}+{\left(\zeta_{\;\bar{Q}}\left(b\right)\right)}^{q}+{\left(\upsilon_{\;\bar{Q}}\left(b\right)\right)}^{q}\leq 1\right)\right)$.

To reduce these limitations, the proposed strategy provides a more flexible environment for decision-makers. By lowering these constraints, decision-makers may try more accurate assessments and render knowledgeable decisions. An overview and comparison of the distinctive features of several techniques, including the recommended one, are given in Table 20.

**Table 20:** A comparison of different approaches' properties.

Methods	Membership degree	Neutral membership degree	Non-membership degree	q
Picture fuzzy ST operators	Yes	Yes	No	No
Spherical fuzzy ST operators	Yes	Yes	No	No
q-spherical fuzzy rough ST operators	Yes	Yes	Yes	Yes

### 5.5 Limitations

Every research endeavor inherently encounters limitations, and the methodology proposed in this study is no different. Hare's discussion highlights these constraints.

1. It's possible that the suggested approaches will only be effective in particular domains or contexts involving decision-making. It's important to understand these limitations and identify the specific situations when the recommended course of action will be most effective.
2. The proposed approach, like any research, is predicated on certain assumptions and simplifications for ease of analysis. These presumptions might not always line up perfectly with actual situations, which could limit how widely or practically the findings can be applied.
3. A case study with four alternatives and four attributes illustrates the effectiveness of the suggested framework. It's crucial to remember that the model is made to support future applications that may require a growth in the number of options and features.
4. The analysis of the alternative's ranking order has been done for certain parameter q values. It is important to note that additional values of these parameters can be explored in terms of ranking order in future studies.

## 6 Conclusion

In this study, we conducted research on aggregation operators, specifically focusing on establishing new sine trigonometric operation laws for Spherical Fuzzy Sets (SFSs). The well-defined operational laws are crucial during decision-making processes, and the sine trigonometric function, with its periodicity and symmetrical nature, is particularly suited to accommodate experts' preferences over multiple periods. We aimed to leverage these characteristics to enhance decision-making and provide a smoother and more impactful decision. We introduced sine trigonometric operation laws for Spherical Fuzzy Numbers (SFNs) and thoroughly studied their properties. By doing so, we developed various average and geometric Aggregation Operators (AOs) based on these laws, providing a framework to combine decision-makers' preferences. We also delved into the elementary relations between the aggregation operators, offering a comprehensive understanding of their interconnections. To apply these newly defined principles to real-world decision-making scenarios, we proposed a novel Multiple Attribute Group Decision-Making (MAGDM) approach for group decision problems. In this approach, goals are categorized in terms of SFNs, and the newly established laws are enforced to guide decision-making. Additionally, we introduced a method to compute the weight of attributes by integrating subjective and objective data measures. To assess the effectiveness of the proposed method, we applied it to a practical example involving the selection of a cloud service provider and a digital transformation vendor for digital transformation. The feasibility and performance of the approach were thoroughly investigated, including a comparative study with existing methodologies to validate its efficacy. It appears that the suggested technique will likely be expanded in the future to include more fields. Among them include resolving multicriteria problems in complicated group decision-making scenarios [30,31], enhancing social trust network integrity [27], and integrating partial language preference connections [28,29] into group decision-making processes. Additionally, we want to extend the scope of the investigation to include several applications related to type 3 fuzzy control, support vector machines, evidence theory [[32], [33], [34]], and other artificial intelligence tools such as neural networks, optimization, feature extraction, and more [35]. In the future, we will use the framework built on new multiple attribute assessment models to tackle fuzziness and ambiguity in a variety of DM parameters, such as design choices, building options, site selection, DM problems, monarch butterfly optimization (MBO) (Feng et al. [36]; Wang et al. [37]; Feng et al. [38,39]), earthworm optimization algorithm (EWA) (Wang et al. [40]), elephant herding optimization (EHO) Wang et al. [41]; Li et al. [42]), moth search (MS) algorithm (Wang [43]), Slime mold algorithm (SMA), and Harris hawks optimization (HHO).

## Funding

This project is funded by 10.13039/501100002383King Saud University, Riyadh, Saudi Arabia.

## Consent for publication

This manuscript has not been published and is not under consideration for publication elsewhere.

## Data availability statement

The accompanying manuscript does not contain any associated data. The paper only presents the written text and does not have any additional data that supports the claims and conclusions presented in the manuscript.

## CRediT authorship contribution statement

**Ahmad Bin Azim:** Conceptualization. **Asad Ali:** Supervision. **Abdul Samad Khan:** Data curation. **Fuad A. Awwad:** Funding acquisition. **Emad A.A. Ismail:** Investigation. **Sumbal Ali:** Software.

## Declaration of competing interest

I am writing to disclose potential conflicts of interest related to the manuscript titled "Utilizing Sine Trigonometric q-Spherical Fuzzy Rough Aggregation Operators for Group Decision-Making and Their Role in Digital Transformation," which I am submitting for consideration for publication in Heliyon Journal.

I, Ahmad Bin Azim, declare that I have no conflicts of interest to report in relation to the research presented in the manuscript titled " Utilizing Sine Trigonometric q-Spherical Fuzzy Rough Aggregation Operators for Group Decision-Making and Their Role in Digital Transformation." All authors have reviewed and agreed with the content of this manuscript.
